# Synthesis, Cytotoxicity and Molecular Docking Studies of the 9-Substituted 5-Styryltetrazolo[1,5-*c*]quinazoline Derivatives

**DOI:** 10.3390/molecules22111719

**Published:** 2017-10-26

**Authors:** Malose J. Mphahlele, Samantha Gildenhuys, Nishal Parbhoo

**Affiliations:** 1 Department of Chemistry, University of South Africa, Private Bag X06, Florida 1710, South Africa; 2 Department of Life & Consumer Sciences, University of South Africa, Private Bag X06, Florida 1710, South Africa; gildes@unisa.ac.za (S.G.); parbhn1@unisa.ac.za (N.P.)

**Keywords:** 9-bromo-5-styryltetrazolo[1,5-*c*]quinazoline, cross-coupling, 9-carbo–substituted 5-styryltetrazolo[1,5-*c*]quinazolines, cytotoxicity, molecular docking

## Abstract

In this paper, we describe the synthesis of the 5-styryltetrazolo[1,5-*c*]quinazolines substituted at the 9-position with a 4-fluorophenyl ring directly or via a conjugated π-spacer (C=C or C≡C bond) based on the 6-bromo-4-chloro-2-styrylquinazoline scaffold. The structures of the synthesized compounds were characterized based on a combination of ^1^H-NMR, ^13^C-NMR, IR and high resolution mass spectral data as well as microanalyses. The tetrazoloquinazolines were evaluated for potential in vitro cytotoxicity against the human breast adenocarcinoma (MCF-7) and cervical cancer (HeLa) cells. The anti-proliferative assays demonstrated that the 9-bromo-5-styryltetrazolo[1,5-*c*]quinazoline **3a** and 9-bromo-5-(4-fluorostyryl)tetrazolo[1,5-*c*]quinazoline **3b** exhibit significant cytotoxicity against both cell lines. A carbon-based substituent at the 9-position resulted in complete loss of cytotoxicity against both cell lines except for the 5,9-bis((*E*)-4-fluorostyryl)tetrazolo[1,5-*c*]quinazoline **4e**, which was found to exhibit comparable cytotoxicity to that of Melphalan (IC_50_ = 61 μM) against the MCF-7 cell line with IC_50_ value of 62 μM. Molecular docking against tubulin (PDB:1TUB) showed that compounds **3a**, **3b** and **4e** bind to the tubulin heterodimer. Binding involves hydrogen bonding for **3a** and **3b** and halogen interactions for **4e**.

## 1. Introduction

**Figure 1:**
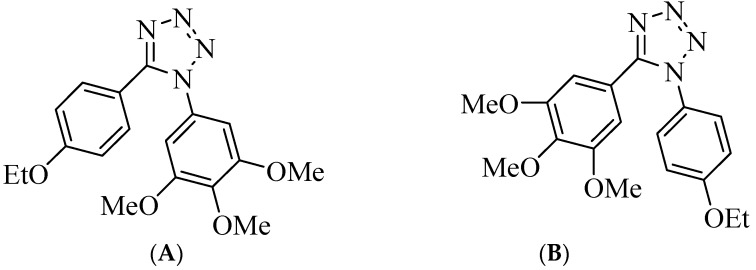
Isomeric 1,5-diaryl substituted 1,2,3,4-tetrazoles (**A**) and (**B**) with antiproliferative and tubulin polymerization inhibition properties.

Nitrogen-based heterocycles such as quinazolines and tetrazoles have earned great interest in medicinal chemistry as anticancer agents [1,2]. Quinazoline derivatives, for example, have been found to inhibit the growth of various cancers including cell carcinoma, bladder, lung, colon, prostate and breast tumour cells [1]. These compounds are known to produce their anticancer activity through inhibition of enzymes such as epidermal growth factor receptor tyrosine kinase (EGFR-TS), dihydrofolate reductase (DHFR), folate thymidylate synthase, tyrosine kinase, aldose reductase, cyclic GMP phosphodiesterase and DNA repairing enzymes [1]. Tetrazole and its derivatives, on the other hand, exhibit a wide variety of biological properties including anti-inflammatory, antibacterial, antifungal, anti-tubercolous, antiviral, antinociceptive, hypoglycaemic, cyclooxygenase inhibitors, and anticancer activities [3,4]. Two series of 1,5-diaryl substituted 1,2,3,4-tetrazoles (**A**) and (**B**) (Figure 1) were synthesized and identified as potent antiproliferative agents and novel tubulin polymerization inhibitors that act at the colchicine site [2]. Colchicine binds tubulin and prevents the formation of microtubules while other anticancer agents can stabilise the tubulin structure, therefore preventing microtubule disassembly [5]. Any disruption in the dynamic nature of microtubule formation and disassociation induces apoptosis [5]. Incorporation of the tetrazole moiety onto the quinazoline framework to comprise the tetrazolo[1,5-*c*]quinazolines has been found to yield derivatives with anti-allergic, bactericide, bronchodilator, anti-ulcer, anti-inflammatory, analgesic and antihypertensive properties [6].

In a previous study from our group, we synthesized series of the 6-bromo- and 6-aryl-2-styrylquinazolin-4(3*H*)-ones and evaluated them for anticancer and antimicrobial properties [7]. In our quest to optimize this class of potential anti-proliferative agents, we decided to modify the 2-styrylquinazolin-4(3*H*)-one framework by (i) annulating a tetrazolo moiety onto the *c*-face and (ii) by varying substituents at the C-6 position to afford novel 5-styryltetrazolo[1,5-*c*]quinazoline derivatives. The main purpose was to prepare the 5-styryltetrazolo[1,5-*c*]quinazolines substituted at the 9 position with a bromine or carbon-based (aryl/styryl/arylethynyl) substituent and to evaluate them for potential anti-proliferative properties. Recourse to the literature revealed three main synthetic approaches towards the tetrazolo[1,5-*c*]quinazolines, which involve either the cyclisation of the 4-hydrazinoquinazolines with nitric acid or nucleophilic aromatic substitution of the 4-chloroquinazolines with sodium azide and subsequent in situ heteroannulation, as well as the cyclization of 5-(2′-aminophenyl)-1*H*-tetrazoles with acetic anhydride [6]. We envisioned that aromatization of the 2-styrylquinazolin-4(3*H*)-ones followed by nucleophilic aromatic substitution-heteroannulation of the intermediate 4-chloro-2-styrylquinazolines with sodium azide would afford the corresponding 5-styryltetrazolo[1,5-*c*]quinazolines. In our view, the presence of a bromine atom in the incipient 9-bromo-5-styryltetrazolo[1,5-*c*]quinazolines would facilitate further transformation via palladium catalysed Suzuki-Miyaura or Sonogashira cross-coupling with arylboronic acids or terminal alkynes, respectively. The potential of these 9-substituted 5-styryltetrazolo[1,5-*c*]quinazolines to bind tubulin and affect changes to the dynamic nature of assembly or disassembly is explained theoretically through molecular docking (in silico) studies.

## 2. Results and Discussion

### 2.1. Chemistry

#### 2.1.1. Synthesis of 9-Bromo-5-styryltetrazolo[1,5-*c*]quinazolines **3a**–**c**

**Scheme 1:**
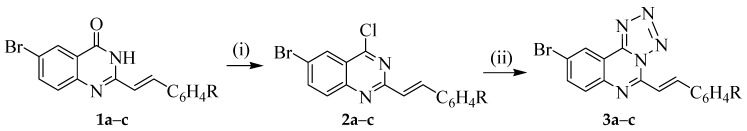
Synthesis of **2a**–**c** and subsequent reaction with sodium azide to afford **3a**–**c**. *Reagents & conditions*: (i) POCl_3_, Et_3_N, reflux, 2 h; (ii) NaN_3_, THF, 45 °C, 4 h.

The 6-bromo-4-chloro-2-styrylquinazolines **2a**–**c** used as substrates in this investigation were prepared by chlorination-dehydration of the corresponding (*E*)-6-bromo-2-styrylquinazolin-4(3*H*)-ones **1a**–**c** [7] with phosphoryl chloride in the presence of triethyl amine under reflux (Scheme 1). The structure of compounds **2a**–**c** was assigned based on their NMR (^1^H and ^13^C in DMSO-*d*_6_), IR and HR-MS data. The ^1^H-NMR spectra of the styrylquinazolines **2a**–**c** (see supplementary material S1) reveal the presence of two doublets in the aromatic region with coupling constant values *J* = 16.0 Hz, which indicates that the double bond exists in *E*-geometry in agreement with the literature assignment for the analogous compounds [8,9]. The 4-chloroquinazolines **2a**–**c** were, in turn, subjected to sodium azide in DMF at 45 °C and we isolated after 4 h the corresponding 5-styryltetrazolo[1,5-*c*]quinazolines **3a**–**c** by aqueous work-up and recrystallization (Scheme 1). Previous studies on the 5-alkyl and 5-aryl substituted tetrazolo[1,5-*c*]quinazolines have revealed that these compounds exist predominantly in the tetrazole form in both the solid state and solution phase [10]. However, the presence of an electron-withdrawing substituent such as a halogen atom in position 8 or 10 was found to result in spontaneous cleavage of the pyrimidine ring of the formed tetrazolo[1,5-*c*]quinazoline into the corresponding *N*-3/5-halogeno-2-(1*H*-tetrazolo-5-yl)phenyl)formamides [11]. The possibility of cleavage of the pyrimidine ring was ruled out by the absence of signal for NH of the tetrazole in the ^1^H-NMR spectra of compounds **3a**–**c** (see supplementary material S1), which typically resonates in the region δ_H_ 16.60–16.90 ppm [11]. The ^13^C-NMR spectra of these products revealed significant downfield shift of signals corresponding to C-6a, C-1a and C-5 to δ 148.7, 143.6 and 137.1 ppm confirming the incorporation of the tetrazolo moiety in analogy with the literature precedent for the 5-unsubstituted tetrazolo[1,5-*c*]quinazoline derivatives [6]. Moreover, IR spectral analysis of **3a**–**c** in the solid state showed the absence of the azide group vibration at about ν_max_ 2100 cm^−1^, which indicates that the compounds exist as the tetrazole form.

A fluorophenyl group on heterocycles has previously been found to enhance biological activity and the lipophilicity of the molecule [8] due to the non-polarizability of the C*sp*^2^-F bond [12]. With the 9-bromo-5-styryltetrazolo[1,5-*c*]quinazolines **3a**–**c** in hand, we decided to attach a 4-fluorophenyl group at the 9-position directly or through a π-spacer (C=C or C≡C bond) to correlate the effect of conjugation to cytotoxic activity in the resultant 9-(4-fluorophenyl/4-fluorostyryl or 4-fluoroethynyl)–substituted 2-styryltetrazoloquinazolines. The transformation was achieved via palladium catalysed Suzuki-Miyaura and Sonogashira cross-coupling reactions as described below.

#### 2.1.2. Suzuki-Miyaura Cross-Coupling in the Synthesis of **4a**–**f**

**Scheme 2:**
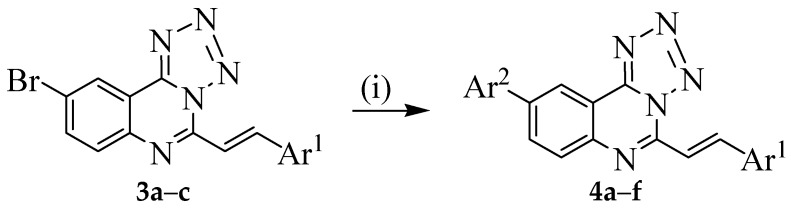
Suzuki-Miyaura cross-coupling of **3a**–**c**. *Reagents & conditions*: (i) ArB(OH)_2_, PdCl_2_(PPh_3_)_2_, K_2_CO_3_, DMF, 100 °C, 3 h.

The Suzuki-Miyaura cross-coupling of **3a**–**c** with either 4-fluorophenyl- or 4-fluorostyrylboronic acid (1.2 equiv.) in the presence of dichlorobis(triphenylphosphine)palladium(II) (PdCl_2_(PPh_3_)_2_) as the source of active Pd(0) species and K_2_CO_3_ (aq) as a base in DMF under reflux afforded the corresponding 9-(4-fluorophenyl/4-fluorostyryl)–substituted 5-tetrazolo[1,5-*c*]quinazolines **4a**–**f** (Table 1, Scheme 2). The latter were also prepared directly from **2a**–**c** via one-pot successive nucleophilic aromatic substitution and heteroannulation with sodium azide under the same reaction conditions described in Scheme 1, followed by the Suzuki-Miyaura cross-coupling with 4-fluorophenylboronic or 4-fluorostyrylboronic acid in the presence of PdCl_2_(PPh_3_)_2_ and K_2_CO_3_ (aq) at 70 °C under argon. The ^1^H- and ^13^C-NMR spectra of compounds **4a**–**f** (see supplementary material S1) are characterized by the presence of an increased number of proton and carbon signals in the aromatic region thus confirming substitution of the bromine atom. The presence of fluorinated aryl groups is further confirmed by the presence of the two sets of intense triplets corresponding to the 4-fluorophenyl group and also the olefinic protons for the 9-(4-fluorostyryl) derivatives **4d**–**f**. The molecular ion region of the mass spectra of these compounds, on the other hand, revealed the absence of the M+ and M+2 peaks in the ratio 1:1 typical for compounds containing the ^79^Br and ^81^Br isotopes, thus confirming their 9-carbosubstituted 5-styryltetrazolo[1,5-*c*]quinazoline nature.

#### 2.1.3. Sonogashira Cross-Coupling of **2a**–**c** and **3a**–**c**

**Scheme 3:**
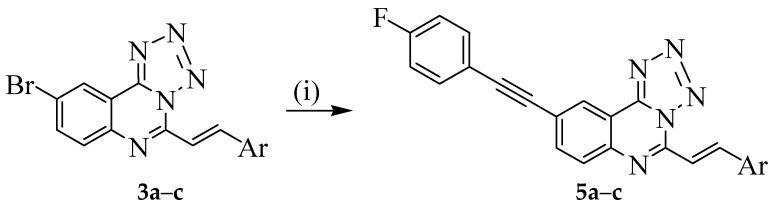
Sonogashira cross-coupling of **3a**–**c**. *Reagents & conditions*: (i) 4-FC_6_H_4_C≡CH, PdCl_2_(PPh_3_)_2_, CuI, Cs_2_CO_3_, DMF, 90 °C, 4 h.

The 9-bromo-5-styryltetrazolo[1,5-*c*]quinazolines **3a**–**c** were also subjected to the Sonogashira cross-coupling with 4-fluorophenylacetylene (1.5 equiv.) as coupling partner in the presence of PdCl_2_(PPh_3_)_2_–CuI catalyst mixture and K_2_CO_3_ as a base at 70 °C for 4 h under argon atmosphere (Table 2, Scheme 3). We isolated by column chromatography on silica gel the corresponding 9-alkynylated styryltetrazolo[1,5-*c*]quinazolines **5a**–**c**. Dechloroamination-cyclization of compounds **2a**–**c** with sodium azide under the same conditions described for the synthesis of **3a**–**c** followed by the Sonogashira cross-coupling of the incipient styryltetrazolo[1,5-*c*]quinazoline with 4-fluorophenylacetylene (1.5 equiv.) in the presence of PdCl_2_(PPh_3_)_2_ and K_2_CO_3_ (aq) at 70 °C for 4 h also afforded compounds **5a**–**d** in a single-pot operation. ^1^H-NMR spectroscopy revealed the presence of two sets of intense symmetrical triplets each integrating for two protons typical of a 4-fluorophenyl group (see supplementary material S1). The presence of the alkynyl group was confirmed by the presence of carbon signals in the region δ 88.0–92.0 ppm of their ^13^C-NMR spectra and the IR absorption bands in the region ν_max_ 2202–2261 cm^−1^. Moreover, the accurate calculated *m*/*z* values for compounds **5a**–**c**, represent in each case a closest fit consistent with the incorporation of a 4-fluorophenylethynyl moiety.

As part of our ongoing research program on polynuclear quinazoline derivatives, which may serve as leads for the design of antitumor agents, we decided to evaluate the 5-styryltetrazolo[1,5-*c*]quinazolines **3**–**5** for potential anti-proliferative properties as described below.

### 2.2. Biological Evaluation

#### 2.2.1. In Vitro Anti-Proliferative Activity of the 5-Styryltetrazolo[1,5-*c*]quinazolines **3**–**5**

The 5-styryltetrazoloquinazolines **3a**–**c**, **4a**–**f** and **5a**–**c** were evaluated for growth inhibitory activity against the human breast cancer (MCF-7) and the human cervical cancer (HeLa) cell lines using the well-established 3-(4,5-dimethylthiazole-2-yl)-2,5-diphenyltetrazoliumbromide based colorimetric cell viability (MTT) assay with Melphalan, a well-known chemotherapeutic compound used as the reference drug. The cytotoxic activities of the tested compounds were expressed as IC_50_ values (the dose that reduces survival to 50%) in μM using Melphalan as a reference standard (Table 3 and Table S4). Within series **3**, only the (*E*)-9-bromo-5-styryltetrazolo[1,5-*c*]quinazoline **3a** and (*E*)-9-bromo-5-(4-fluorostyryl)tetrazolo[1,5-*c*]quinazoline **3b** were found to exhibit significant cytotoxicity against both cell lines. 9-Bromo-5-styryltetrazolo[1,5-*c*]quinazoline **3a** represents the most cytotoxic compound and is considerably more cytotoxic than the anticancer drug Melphalan against both the MCF-7 and HeLa cell lines. No activity was observed for the 4-chlorostyryl derivative **3c**. Replacement of bromine atom with a 4-fluorophenyl group in compounds **4a**–**c** resulted in loss of activity against both cell lines. Within the 9-styryl substituted derivatives **4d**–**f**, only the 9-(4-fluorostyryl) derivative **4e** was found to be selective against the MCF-7 cell line and to exhibit comparable cytotoxicity to that of Melphalan (IC_50_ = 61 μM) with IC_50_ value of 62 μM. Replacement of bromine with 4-fluorophenylethynyl group in compounds **5a**–**c** also resulted in complete loss of activity against both cell lines.

Previous structure activity relationship studies revealed that the entire styrylquinazolinone scaffold was essential for the inhibition of tubulin polymerization and their biological activity is enhanced by the presence of a halide or small hydrophobic substituents at position 6 [13]. From these preliminary in vitro cytotoxicity results and SAR, it is observed that a bulky group at the 9-position of the 5-styryltetrazolo[1,5-*c*]quinazoline moiety generally leads to loss of cytotoxicity. The observed cytotoxicity of **3a** and **3b** is due to the presence of a halide or small hydrophobic substituents at position 9 in analogy with the literature observation for the analogous styrylquinazolinones [7,13,14]. The 6-substituted 2-styrylquinazoline derivatives have been found to exhibit antimitotic properties and to inhibit tubulin polymerization [13]. This protein is often used as a model in molecular docking studies to predict the hypothetical protein-ligand binding mode, which plays a significant role in structural based drug design and structure activity relationship. Molecules that bind to tubulin are known to prevent the controlled assembly and disassembly of the microtubule structures and induce apoptosis [5]. The anticancer properties of the 6-substituted 2-styrylquinazolines [13] and the 1,5-diaryl substituted 1,2,3,4-tetrazoles [2] as tubulin polymerization inhibitors prompted us to conduct molecular docking of the most active compounds **3a**, **3b** and **4e** against tubulin to predict their hypothetical protein-ligand binding mode.

#### 2.2.2. Molecular Docking Studies of **3a**, **3b** and **4e** into Tubulin

**Figure 2:**
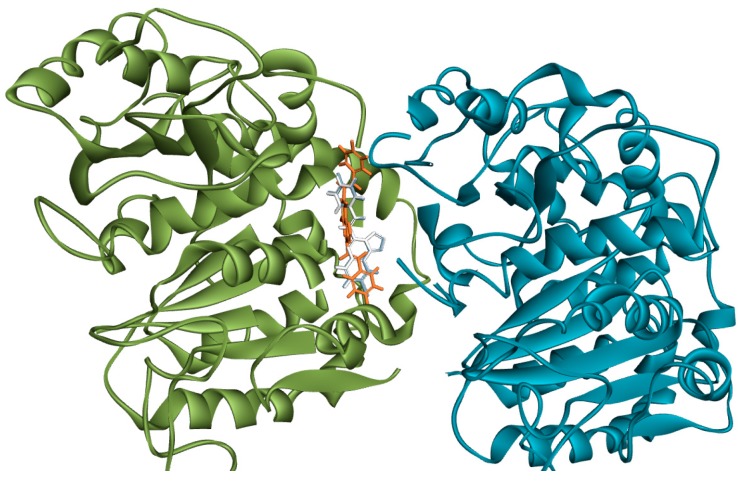
Compounds **3a**, **3b** (light and dark grey respectively) and **4e** (orange) docked at the dimer interface of a tubulin heterodimer (PDB code 1TUB) with chains coloured blue and green.

**Figure 3:**
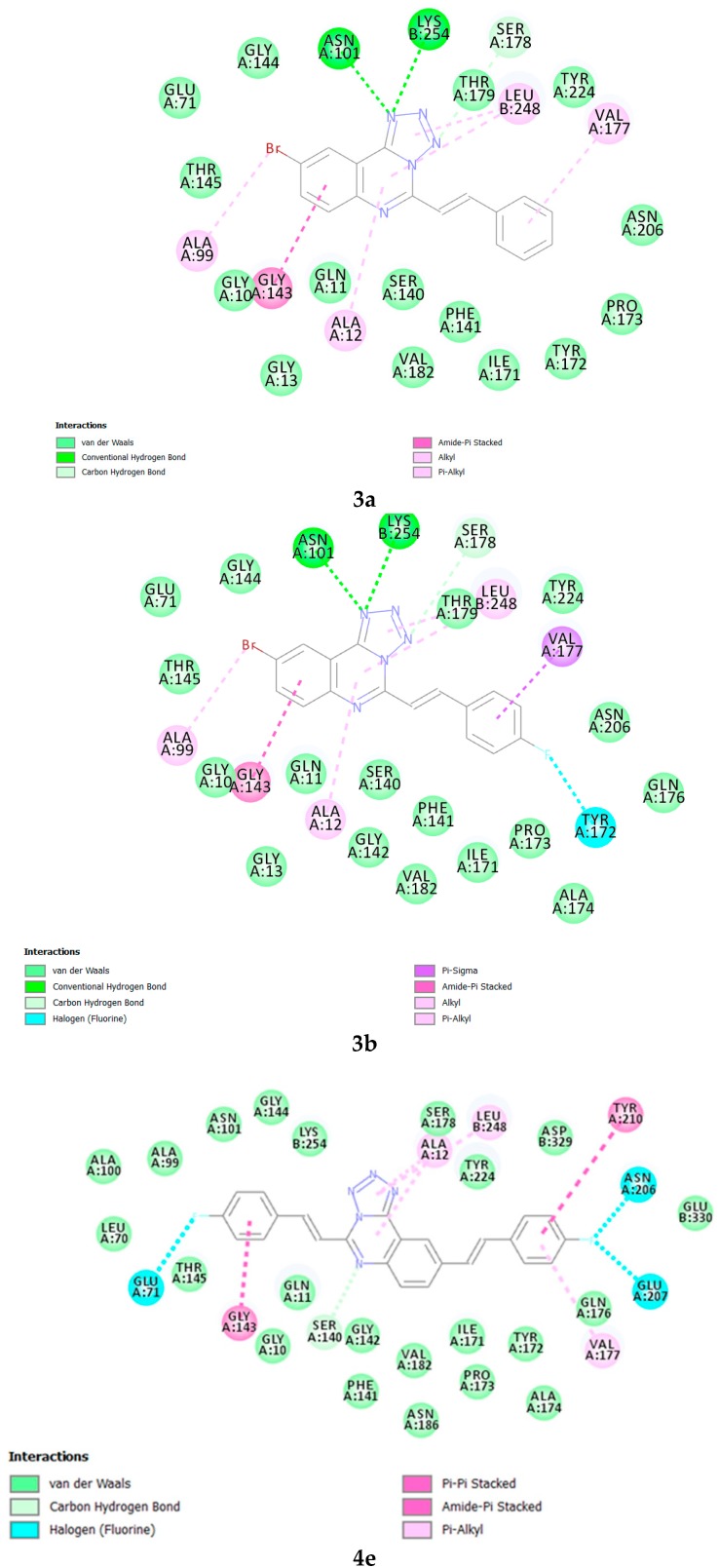
2D interaction diagram for the binding of tubulin (PDB code 1TUB) with **3a**, **3b** and **4e**. Residues are annotated with their 3-letter amino acid code and interactions listed below the diagram.

A crystal structure of tubulin was obtained from the protein data bank (PDB ID: 1TUB). Molecular docking was conducted in order to determine if compounds **3a**, **3b** and **4e** could bind tubulin. The 6-bromo–substituted derivative **3a** docked to tubulin between the two subunits of the heterodimer (Figure 2) and binding interactions included a hydrogen bond between the derivative and ASN101 or LYS254 of tubulin as well as pi-alkyl, amide-pi stacked and alkyl interactions (Figure 3). Derivative **3b** also bound tubulin in the same site between the dimeric subunits with a hydrogen bond that forms between ASN101 or LYS254 and a halogen bond between the derivative’s fluorine residue and TYR172 of tubulin (Figure 3). When **4e** was docked to the tubulin heterodimer (Figure 3) it also bound the same site, but the binding did not involve hydrogen bonding but rather the presence of two fluorine residues resulted in halogen interactions between the compound and GLU71, ASN206 and GLU207 of tubulin. Docking studies revealed that the tetrazoloquinazolines that showed anti-proliferative activity could act by binding tubulin at the dimer interface. This binding will affect the microtubule changes that take place in cells during cell division and hence lead to cell death.

## 3. Experimental

Melting points were recorded on a Thermocouple digital melting point apparatus (Stuart, Staffordshire, UK) and are uncorrected. IR spectra were recorded as powders using a Bruker VERTEX 70 FT-IR Spectrometer (Bruker Optics, Billerica, MA, USA) with a diamond ATR (attenuated total reflectance) accessory by using the thin-film method. For column chromatography, Merck kieselgel 60 (0.063–0.200 mm) (Merck KGaA, Frankfurt, Germany) was used as the stationary phase. NMR spectra were obtained as DMSO-*d*_6_ solutions using either the Varian 300 MHz NMR spectrometer (Varian Inc., Palo Alto, CA, USA) or Agilent 500 MHz NMR spectrometer (Agilent Technologies, Oxford, UK) and the chemical shifts are quoted relative to the TMS peak. Low- and high-resolution mass spectra were recorded at an ionization potential of 70 eV using Waters Synapt G2 Quadrupole Time-of-flight mass spectrometer (Waters Corp., Milford, MA, USA) at the University of Stellenbosch Mass Spectrometry Unit. The elemental analyses were obtained using a Vario EL Cube Elemental Analyzer (Elementar, Langenselbold, Germany) at the University of Stellenbosch Central Analytical Facility. The synthesis and characterization of compounds **1a**–**c** have been described before [7].

### 3.1. Typical Procedure for the Phosphoryl-Mediated Chlorination-Dehydration of ***1a**–**c***; the Synthesis of ***1a**–**c***

A mixture of 6-bromo-2-styrylquinazolin-4(3*H*)-one **1a** (0.50 g, 1.55 mmol), phosphoryl chloride (20 mL) and triethylamine (2 drops) was heated under reflux for 8 h and then allowed to cool to RT. The mixture was poured slowly into an ice-cold aqueous ammonia with vigorous stirring. The resulting precipitate was filtered, washed thoroughly with ice-cold water and then recrystallized to afford **2**. Compounds **2a**–**c** were prepared in this fashion.

*(E)-6-Bromo-4-chloro-2-styrylquinazoline* (**2a**). Yellow solid (0.41 g, 78%), m.p. 203–205 °C (ethanol); ν_max_ (ATR) 539, 682, 752, 839, 972, 1557, 3934 cm^−1^; ^1^H-NMR (500 MHz, DMSO-*d*_6_) 7.27 (1H, d, *J*_trans_ = 16.0 Hz, H_b_), 7.36–7.44 (3H, m, 3′,5′-H and 4′-H), 7.65 (2H, d, *J* = 8.7 Hz, 2′,6′-H), 7.85 (1H, d, *J* = 8.7 Hz, 8-H), 7.98 (1H, dd, *J* = 2.4 Hz and 8.7 Hz, 7-H), 8.13 (1H, d, *J*_trans_ = 16.0 Hz, H_a_), 8.35 (1H, d, *J* = 2.4 Hz, 5-H); ^13^C-NMR (125 MHz, DMSO-*d*_6_) 119.3, 120.2, 122.9, 128.3, 128.6, 129.0, 129.6, 130.7, 135.1, 138.0, 140.4, 147.1, 153.0, 161.0; HRMS (ES): MH^+^, found 344.9785. C_16_H_11_N_2_^35^Cl^79^Br^+^ requires 344.9794. Anal. calcd. for C_16_H_10_N_2_ClBr: C, 55.60; H, 2.92; N, 8.11. Found: C, 55.63; H, 2.90; N, 8.06.

*(E)-6-Bromo-4-chloro-2-(4-fluorostyryl)quinazoline* (**2b**). Yellow solid (0.37 g, 71%), m.p. 215–217 °C (ethanol); ν_max_ (ATR) 520, 819, 828, 948, 1208, 1598, 2922 cm^−1^; ^1^H-NMR (300 MHz, DMSO-*d*_6_) 7.10 (2H, t, *J* = 9.0 Hz, 3′,5′-H), 7.20 (1H, d, *J*_trans_ = 16.0 Hz, H_b_), 7.63 (2H, dd, *J =* 2.0 Hz and 9.0 Hz, 2′,6′-H), 7.85 (1H, d, *J* = 8.7 Hz, 8-H), 8.00 (1H, dd, *J* = 2.4 Hz and 8.7 Hz, 7-H), 8.10 (1H, d, *J*_trans_ = 16.0 Hz, H_a_), 8.37 (1H, d, *J* = 2.4 Hz, 5-H); ^13^C-NMR (75 MHz, DMSO-*d*_6_) 166.6 (d, ^2^*J*_CF_ = 20.9 Hz), 119.2, 120.4, 123.0, 128.5, 129.2, 130.45 (d, ^3^*J*_CF_ = 8.5 Hz), 131.8 (d, ^4^*J*_CF_ = 3.8 Hz), 137.9, 138.8, 147.5, 152.6, 160.9, 163.5 (d, ^1^*J*_CF_ = 247.5 Hz); HRMS (ES): MH^+^, found 362.9698. C_16_H_10_N_2_F^35^Cl^79^Br^+^ requires 362.9700. Anal. calcd. for C_16_H_9_N_2_FClBr: C, 52.85; H, 2.49; N, 7.70. Found: C, 52.83; H, 2.47; N, 7.68.

*(E)-6-Bromo-4-chloro-2-(4-chlorostyryl)quinazoline* (**2c**). Yellow solid (0.47 g, 71%), m.p. 210–212 °C (ethanol); ν_max_ (ATR) 496, 662, 813, 830, 972, 991, 1558, 2921 cm^−1^; ^1^H-NMR (300 MHz, DMSO-*d*_6_) 7.24 (1H, d, *J*_trans_ = 16.0 Hz, H_b_), 7.38 (2H, d, *J* = 8.0 Hz, 3′,5′-H), 7.58 (2H, d, *J* = 8.7 Hz, 2′,6′-H), 7.85 (1H, d, *J* = 9.0 Hz, 8-H), 7.99 (1H, dd, *J* = 2.4 Hz and 9.0 Hz, 7-H), 8.08 (1H, d, *J*_trans_ = 16.0 Hz, H_a_), 8.37 (1H, d, *J* = 2.4 Hz, 5-H); 13C-NMR (125 MHz, DMSO-*d*_6_) 119.3, 123.3, 124.4, 128.0, 128.3, 128.4, 130.0, 130.6, 131.7, 133.2, 133.9, 134.4, 137.8, 148.2; HRMS (ES): MH^+^, found: 378.9492. C_16_H_10_N_2_^35^Cl^79^Br^+^ requires: 378.9404. Anal. calcd. for C_16_H_9_N_2_Cl_2_Br: C, 50.56; H, 2.39; N, 7.37. Found: C, 50.54; H, 2.36; N, 7.36.

### 3.2. Typical Procedure for the Synthesis of Tetrazoloquinazolines ***3a**–**c***

A stirred mixture of **2** (1 equivalent) and sodium azide (1.2 equivalent) in DMF (26 mL/mmol of **2**) was heated at 45 °C for 4 h. The mixture was allowed to cool to RT and then quenched with ice-cold water. The resultant precipitate was filtered and recrystallized from acetonitrile to afford **3**. The following compounds were prepared in this fashion:

*(E)-9-Bromo-5-styryltetrazolo[1,5-c]quinazoline* (**3a**). A mixture of **2a** (0.20 g, 0.58 mmol) and sodium azide (0.04 g, 0.62 mmol) in DMF (15 mL) afforded **3a** as a brown solid (0.15 g, 72%), m.p. 214–216 °C (acetonitrile); ν_max_ (ATR) 558, 686, 761, 836, 975, 1076, 1385, 1495, 1633, 3026, 3082 cm^−1^; ^1^H-NMR (300 MHz, DMSO-*d*_6_) 7.48–7.50 (3H, m, 3′,5′-H and 4′-H), 7.89 (2H, d, *J* = 8.4 Hz, 2′,6′-H), 7.91 (1H, d, *J*_trans_ = 16.0 Hz, H_b_), 8.06 (1H, d, *J* = 8.7 Hz, 7-H ), 8.19 (1H, dd, *J* = 2.4 and 9.0 Hz, 8-H), 8.37 (1H, d, *J*_trans_ = 16.0 Hz, H_a_), 8.67 (1H, d, *J* = 2.4 Hz, 10-H); ^13^C-NMR (75 MHz, DMSO-*d*_6_) 116.5, 116.9, 122.3, 127.0, 129.1, 129.7, 131.0, 131.3, 135.0, 137.1, 142.2, 143.0, 143.6, 149.0; HRMS (ES): MH^+^, found: 352.0189. C_16_H_11_N_5_^79^Br^+^ requires: 352.0188. Anal. calcd. for C_16_H_10_N_5_Br: C, 54.56; H, 2.86; N, 19.89. Found: C, 54.54; H, 2.89, N, 9.86.

*(E)-9-Bromo-5-(4-fluorostyryl)tetrazolo[1,5-c]quinazoline* (**3b**). A mixture of **2b** (0.20 g, 0.55 mmol) and sodium azide (0.04 g, 0.66 mmol) in DMF (14 mL) afforded **3b** as a brown solid (0.16 g, 81%), m.p. 217–218 °C (acetonitrile); ν_max_ (ATR) 517, 828, 979, 1079, 1161, 123, 1269, 1470, 1508, 1586, 1637 cm^−1^; ^1^H-NMR (300 MHz, DMSO-*d*_6_) 7.34 (2H, t, *J* = 9.0 Hz, 3′,5′-H), 7.89 (1H, d, *J*_trans_ = 16.0 Hz, H_b_), 8.00–8.07 (3H, m, Ar), 8.19 (1H, dd, *J* = 2.4 and 9.0 Hz, 8-H), 8.38 (1H, d, *J*_trans_ = 16.0 Hz, H_a_), 8.69 (1H, d, *J* = 2.4 Hz, 10-H); ^13^C-NMR (75 MHz, DMSO-*d*_6_) 116.4, 166.6 (d, ^2^*J*_CF_ = 21.7 Hz), 116.7, 122.2, 126.7, 130.7, 131.5 (d, ^3^*J*_CF_ = 8.5 Hz), 131.67 (d, ^4^*J*_CF_ = 2.8 Hz), 137.2, 141.8, 142.2, 143.6, 148.7, 163.9 (d, ^1^*J*_CF_ = 247.4 Hz); HRMS (ES): MH^+^, found: 370.0101. C_16_H_10_N_5_F^79^Br^+^ requires: 370.0104. Anal. calcd. for C_16_H_9_N_5_FBr: C, 51.91; H, 2.45; N, 18.92. Found: C, 51.90; H, 2.42; N, 18.89.

*(E)-9-Bromo-5-(4-chlorostyryl)tetrazolo[1,5-c]quinazoline* (**3c**). A mixture of **2c** (0.20 g, 0.63 mmol) and sodium azide (0.05 g, 0.76 mmol) in DMF (20 mL) afforded **3c** as a brown solid (0.15 g, 63%), m.p. 234–236 °C (acetonitrile); ν_max_ (ATR) 465, 500, 766, 809, 820, 1090, 1387, 1470, 1501, 1582, 1633 cm^−1^; ^1^H-NMR (300 MHz, DMSO-*d*_6_) 7.44 (2H, d, *J* = 8.0 Hz, 3′,5′-H), 7.93 (1H, d, *J*_trans_ = 16.0 Hz, H_b_), 7.91 (2H, d, *J* = 7.5 Hz, 2′,6′-H), 8.05 (1H, d, *J* = 8.0 Hz, 7-H), 8.17 (1H, d, *J* = 7.5 Hz, 8-H), 8.36 (1H, d, *J*_trans_ = 16.0 Hz, H_a_), 8.70 (1H, d, *J* = 2.4 Hz, 10-H); ^13^C-NMR (75 MHz, DMSO-*d*_6_) 116.7, 117.3, 122.4, 126.7, 129.7 (2 × C), 130.8, 133.9, 135.8, 137.1, 141.5, 142.2, 143.5, 148.9; RMS (ES): MH^+^, found: 386.1464. C_16_H_9_N_5_^35^Cl^79^Br ^+^ requires: 386.1464. Anal. calcd. for C_16_H_9_N_5_ClBr: C, 49.70; H, 2.35; N, 18.11. Found: C, 49.67; H, 2.33; N, 18.10.

### 3.3. Typical Procedure for the Suzuki-Miyaura Cross-Coupling of ***3a**–**c***; Synthesis of ***4a**–**f***

To a three-necked flask equipped with stirrer, rubber septum, thermometer and a condenser fitted with a balloon at the top was added **3a** (0.30 g, 0.85 mmol) and DMF (15 mL) under nitrogen atmosphere. 4-Fluorophenylboronic acid (0.18 g, 1.28 mmol), PdCl_2_(P(Cy)_3_)_2_ (0.06 g, 0.085 mmol), K_2_CO_3_ (0.14 g, 1.02 mmol) and water (5 mL) were added to the mixture under nitrogen atmosphere. The mixture was heated at 100 °C for 3 h and then poured into ice-cold water. The product was extracted into chloroform and the combined organic phases were washed with brine, dried over MgSO_4_ and the salt was filtered off. The solvent was evaporated under reduced pressure and the residue was purified by column chromatography on silica gel using 9:1 toluene-ethyl acetate (*v*/*v*) as an eluent. The following products were prepared in this fashion.

*(E)-9-(4-Fluorophenyl)-5-styryltetrazolo[1,5-c]quinazoline* (**4a**). Solid (0.17 g, 53%), *R_f_* 0.62, m.p. 243–245 °C; ν_max_ (ATR) 524, 588, 683, 749, 823, 972, 1227, 1484, 1634, 2854, 2924, 3029 cm^−1^; δ_H_ (500 MHz, DMSO-*d*_6_) 7.37 (3H, t, *J* = 9.0 Hz, 2″,6″-H), 7.47–7.51 (3H, m, Ph), 7.90–7.99 (5H, m, H_a_, 2″,6″-H and Ph), 8.18 (1H, d, *J* = 9 Hz, 7-H), 8.32 (1H, dd, *J* = 2.0 Hz and 8.0 Hz, 8-H), 8.37 (1H, d, *J*_trans_ = 16.0 Hz, H_b_), 8.73 (1H, d, *J* = 2.0 Hz, 10-H); 13C-NMR (125 MHz, DMSO-*d*_6_) 115.6, 116.6 (^2^*J*_CF_ = 21.9 Hz), 116.8, 121.7, 129.0, 129.4, 129.7 (2 × C), 129.9 (^3^*J*_CF_ = 8.5 Hz), 131.2, 132.7, 135.1, 140.0, 142,4, 142.5 (^3^*J*_CF_ = 2.9 Hz), 143.1, 149.6, 163.0 (^1^*J*_CF_ = 244.6 Hz); ^19^F-NMR (282 MHz, DMSO-*d*_6_) −113.08 (1F, ddd, *J* = 8.0, 14.6, 21.2 Hz); HRMS (ES): MH^+^, found: 368.1311. C_22_H_14_N_5_F^+^ requires: 368.1307. Anal. calcd. for C_16_H_14_N_5_F: C, 71.92; H, 3.84; N, 19.06. Found: C, 71.91; H, 3.82; N, 19.03.

*(E)-9-(4-Fluorophenyl)-5-(4-fluorostyryl)tetrazolo[1,5-c]quinazoline* (**4b**). Solid (0.22 g, 51%), *R_f_* 0.84, m.p. 218–220 °C; ν_max_ (ATR) 537, 820, 975, 1158, 1234, 1484, 1511, 1635, 2853, 2924, 3045 cm^−1^; δ_H_ (500 MHz, DMSO-*d*_6_) 7.33 (2H, t, *J* = 7.5 Hz, 3′,5′-H), 7.37 (2H, t, *J* = 7.5 Hz, 3″,5″-H), 7.88 (1H, d, *J*_trans_ = 16.0 Hz, H_b_), 7.96 (2H, t, *J* = 7.5 Hz, 2′,4′-H), 8.00 (2H, t, *J* = 7.5 Hz, 2″,6″-H), 8.17 (1H, d, *J* = 8.5 Hz, 7-H), 8.32 (1H, dd, *J* = 2 and 8.5 Hz, 8-H), 8.36 (1H, d, *J*_trans_ = 16.0 Hz, H_a_), 8.71 (1H, d, *J* = 2.0 Hz, 10-H); ^13^C-NMR (125 MHz, DMSO-*d*_6_) 115.6, 116.6 (^2^*J*_CF_ = 21.8 Hz), 116.7 (^2^*J*_CF_ = 21.8 Hz), 116.8, 121.7, 129.4, 129.9 (^3^*J*_CF_ = 8.5 Hz), 131.4 (^3^*J*_CF_ = 8.5 Hz), 131.8 (^3^*J*_CF_ = 2.8 Hz), 132.6, 135.0 (^3^*J*_CF_ = 2.9 Hz), 140.0, 141.3, 142.5 (^3^*J*_CF_ = 2.9 Hz), 143.0, 149.6, 163.0 (^1^*J*_CF_ = 245.6 Hz), 163.9 (^1^*J*_CF_ = 247.5 Hz); ^19^F-NMR (282 MHz, DMSO-*d*_6_) −109.4 (1F, ddd, *J* = 4.7, 8.0 and 19.7 Hz), −133.74 (1F, ddd, *J* = 4.7, 8.0 and 19.8 Hz); HRMS (ES): MH^+^, found: 386.1214. C_22_H_14_N_5_F_2_^+^ requires 386.1217. Anal. calcd. for C_16_H_13_N_5_F_2_: C, 68.57; H, 3.40; N, 18.17. Found: C, 68.56; H, 3.37; N, 18.16.

*(E)-5-(4-Chlorostyryl)-9-(4-fluorophenyl)tetrazolo[1,5-c]quinazoline* (**4c**). Solid (0.11 g, 50%), *R_f_* 0.56, m.p. 238–240 °C; ν_max_ (ATR) 473, 510, 766, 829, 981, 1074, 1296, 1387, 1469, 1501, 1582, 1633, 3069 cm^−1^; δ_H_ (500 MHz, DMSO-*d*_6_) 7.03 (1H, d, *J* = 16.5 Hz, H_b_), 7.32 (2H, t, *J* = 9.0 Hz, 3′,5′-H), 7.52 (2H, d, *J* = 9.0 Hz, 3″,5″-H), 7.68 (2H, d, *J =* 9.0 Hz, 2′,6′-H), 7.76 (1H, d, *J* = 8.0 Hz, 7-H), 8.82 (2H, t, *J* = 9.0 Hz, 2″,6″-H), 7.51 (1H, d, *J*_trans_ = 16.5 Hz, H_a_), 8.12 (1H, dd, *J* = 2.0 Hz and 8.0 Hz, 8-H), 8.29 (1H, d, *J* = 2.0 Hz, 10-H); ^13^C-NMR (125 MHz, DMSO-*d*_6_) 116.1 (d, ^2^*J*_CF_ = 21.8 Hz), 121.1, 121.8, 124.2, 127.7, 128.1, 128.9, 129.4 (^3^*J*_CF_ = 8.5 Hz), 129.6, 130.4, 132.6, 133.9 (d, _4_*J*_CF_ = 2.9 Hz), 134.0, 135.4, 135.8, 139.1, 151.7, 163.3 (^1^*J*_CF_ = 243.6 Hz); ^19^F-NMR (282 MHz, DMSO-*d*_6_) −113.7 (1F, ddd, *J* = 6.6, 9.9 and 19.7 Hz); HRMS (ES): MH^+^, found: 402.0897. C_24_H_14_N_5_F^35^Cl^+^ requires 402.0922. Anal. calcd. for C_16_H_13_N_5_F_2_: C, 65.76; H, 3.26; N, 17.43. Found: C, 65.73; H, 3.25; N, 17.42.

*9-((E)-4-Fluorostyryl)-5-((E)-styryl)tetrazolo[1,5-c]quinazoline* (**4d**). Solid (0.15 g, 47%), *R_f_* 0.51, m.p. 213–215 °C; ν_max_ (ATR) 520, 591, 691, 754, 832, 980, 1235, 1501, 1634, 3025 cm^−1^; δ_H_ (500 MHz, DMSO-*d*_6_) 7.24 (2H, t, *J* = 8.5 Hz, 3″,5″-H), 7.37 (1H, t, *J* = 5.0 Hz, 4′), 7.38 (1H, d, *J*_trans_ = 16.0 Hz, H_b_), 7.50 (2H, d, *J* = 7.0 Hz, 3′,5′-H), 7.52 (1H, d, *J*_trans_ = 16.0 Hz, H_b_), 7.58 (1H, d, *J*_trans_ = 16.0 Hz, H_a_), 7.73 (2H, t, *J* 7.0 = Hz, 2′,6′-H), 7.89 (2H, t, *J* = 7.0 Hz, 2″,6″-H), 8.10 (1H, d, *J* = 8.5 Hz, 7-H), 8.27 (1H, dd, *J* = 1.5 Hz and 8.5 Hz, 8-H), 8.35 (1H, d, *J*_trans_ = 16.0 Hz, H_a_), 8.69 (1H, d, *J* = 1.5 Hz, 10-H); ^13^C-NMR (125 MHz, DMSO-*d*_6_) 114.9, 116.5 (d, ^2^*J*_CF_ = 20.8 Hz), 129.1, 129.3, 129.4, 129.5, 129.6, 129.9 (2 × C), 130.0 (d, ^2^*J*_CF_ = 20.9 Hz), 130.6, 132.0, 132.9 (d, ^4^*J*_CF_ = 2.8 Hz), 134.9, 137.2, 140.6, 141.6, 146.8, 148.7, 163.0 (d, ^1^*J*_CF_ = 245.6 Hz); ^19^F-NMR (282 MHz, DMSO-*d*_6_) −113.71 (1F, ddd, *J* = 6.6, 9.9, 19.7 Hz); HRMS (ES): MH^+^, found: 394.1468. C_24_H_17_N_5_F^+^ requires: 394.1455. Anal. calcd. for C_16_H_16_N_5_F_2_: C, 73.27; H, 4.10; N, 17.80. Found: C, 73.24; H, 4.07; N, 17.78.

*5,9-Bis((E)-4-fluorostyryl)tetrazolo[1,5-c]quinazoline* (**4e**). Solid (0.25 g, 58%), *R_f_* 0.80, m.p. 241–243 °C; ν_max_ (ATR) 508, 819, 971, 1159, 1233, 1484, 1509, 1635, 2853, 2924, 3045 cm^−1^; δ_H_ (300 MHz, DMSO-*d*_6_) 7.22 (2H, t, *J* = 9.0 Hz, 3′,5′-H), 7.30 (2H, t, *J* = 9.0 Hz, 3″,5″-H), 7.45 (1H, d *J*_trans_ = 16.0 Hz, H_b_), 7.54 (1H, d, *J*_trans_ = 16.0 Hz, H_b_), 7.69 (2H, t, *J* = 7.5 Hz, 2′,6′-H), 7.80 (1H, d, *J*_trans_ = 16.0 Hz, H_a_), 7.96 (2H, t, *J* = 7.5 Hz, 2″,6″-H), 8.03 (1H, d, *J* = 8.5 Hz, 7-H), 8.21 (1H, dd, *J* = 1.5 and 8.5 Hz, 8-H), 8.28 (1H, d, *J*_trans_ = 16.0 Hz, H_a_), 8.58 (1H, d, *J* = 1.5 Hz, 10-H); ^13^C-NMR (75 MHz, DMSO-*d*_6_) 115.5, 116.1 (^2^*J*_CF_ = 20.9 Hz), 116.6 (^2^*J*_CF_ = 21.9 Hz), 122.0, 127.1, 128.8, 129.0 (d, ^3^*J*_CF_ = 8.6 Hz), 130.9, 131.3, 131.5 (d, ^3^*J*_CF_ = 8.5 Hz), 131.6, 131.8 (d, ^4^*J*_CF_ = 2.8 Hz), 133.7 (d, ^4^*J*_CF_ = 3.0 Hz), 138.4, 141.0, 142.5, 142.6, 149.5, 162.0 (d, ^1^*J*_CF_ = 248.0 Hz), 163.8 (d, ^1^*J*_CF_ = 247.4 Hz); ^19^F-NMR (282 MHz, DMSO-*d*_6_) −109.5 (1F, ddd, *J* = 5.2, 8.5 and 18.3 Hz), −110.2 (1F, ddd, *J* = 5.2, 9.8 and 15.0 Hz); HRMS (ES): MH^+^, found: 412.1366. C_24_H_16_N_5_^35^F_2_^+^ requires 412.1374. Anal. calcd. for C_16_H_15_N_5_F_2_: C, 70.07; H, 3.67; N, 17.02. Found: C, 70.05; H, 3.66; N, 17.00.

*5-((E)-4-Chlorostyryl)-9-((E)-4-fluorostyryl)tetrazolo[1,5-c]quinazoline* (**4f**). Solid (0.12 g, 55%), *R_f_* 0.51, m.p. 238–241 °C; ν_max_ (ATR) 515, 592, 789, 814, 840, 970, 1014, 1074, 1096, 1234, 1499, 1589, 1636 cm^−1^; δ_H_ (500 MHz, DMSO-*d*_6_) 7.22 (2H, t, *J* = 7.5 Hz, 3″,5″-H), 7.45 (1H, d, *J*_trans_ = 16.0 Hz, H_b_), 7.51 (2H, d, *J* = 7.5 Hz, 3′,5′-H), 7.54 (1H, d, *J*_trans_ = 16.0 Hz, H_b′_), 7.70 (2H, t, *J* = 7.5 Hz, 2′,4′-H), 7.86 (1H, d, *J*_trans_ = 16.0 Hz, H_a_), 7.90 (2H, d, *J* = 7.5 Hz, 2″,6-H), 8.04 (1H, d, *J* = 8.5 Hz, 7-H), 8.23 (1H, dd, *J* = 1.5 and 8.5 Hz, 8-H), 8.28 (1H, d, *J*_trans_ = 16.0 Hz, H_a′_), 8.59 (1H, d, *J* 1.5 Hz, 10-H); ^13^C-NMR (125 MHz, DMSO-*d*_6_) 116.3 (d, ^2^*J*_CF_ = 21.7 Hz), 121.4, 123.4, 127.7, 129.2 (d, ^3^*J*_CF_ = 8.0 Hz), 129.7, 129.5, 133.1, 135.8 (d, ^4^*J*_CF_ = 3.0 Hz), 136.9, 130.4, 132.6, 133.9 (d, _4_*J*_CF_ = 2.9 Hz), 134.0, 135.4, 135.8, 139.1, 148.6, 154.9, 163.1 (d, ^1^*J*_CF_ = 242.5 Hz); ^19^F-NMR (282 MHz, DMSO-*d*_6_) −113.71 (1F, ddd, *J* = 6.6, 10.0, 19.7 Hz); HRMS (ES): MH^+^, found: 428.1073. C_24_H_16_N_5_^35^ClF^+^ requires: 428.1078. Anal. calcd. for C_16_H_15_N_5_FCl, 67.37; H, 3.53; N, 16.37. Found: C, 67.35; H, 3.49; N, 16.34.

### 3.4. Typical Procedure for the One-Pot S_N_Ar-Heteroannulation and Suzuki-Miyaura Cross-Coupling of ***2a**–**c*** to Afford ***4a**–**f***

A mixture of **2a** (0.20 g, 0.58 mmol) and sodium azide (0.04 g, 0.62 mmol) was stirred at 45 °C for 4 h. 4-Fluorophenylboronic acid (0.12 g, 0.86 mmol), PdCl_2_(P(Cy)_3_)_2_ (0.04 g, 0.058 mmol), K_2_CO_3_ (1.00 g, 0.70 mmol) and water (15 mL) were then added to the mixture under nitrogen atmosphere. The mixture was heated at 100 °C for 3 h and then poured into ice-cold water. The product was extracted into chloroform and the combined organic phases were washed with brine, dried over MgSO_4_ and the salt was filtered off. The solvent was evaporated under reduced pressure and the residue was purified by column chromatography on silica gel. Compounds **4a**–**f** were prepared in this fashion; see Table 1 under Scheme 2 for the corresponding yields.

### 3.5. Typical Procedure for the Sonogashira Cross-Coupling of ***3a**–**c***; Synthesis of ***5a**–**c***

A mixture of **3a** (0.30 g, 0.86 mmol), 4-fluorophenylacetylene (0.16 g, 1.30 mmol), PdCl_2_(P(Cy)_3_)_2_ (0.06 g, 0.08 mmol), CuI (0.016 g, 0.08 mmol) and Cs_2_CO_3_ (0.40 g, 0.13 mmol) in DMF (15 mL) was heated at 100 °C for 2 h under nitrogen atmosphere. The mixture was then poured into ice-cold water. The resultant precipitate was filtered and purified by column chromatography on silica gel using 9:1 toluene–ethyl acetate (*v*/*v*) as an eluent. Compounds **5a**–**c** were prepared in this fashion.

*(E)-9-((4-Fluorophenyl)ethynyl)-5-styryltetrazolo[1,5-c]quinazoline* (**5a**). Solid (0.17 g, 50%), *R_f_* 0.70, m.p. 175–177 °C; ν_max_ (ATR) 467, 686, 750, 829, 975, 1229, 1506, 1588, 1630, 2202, 2923 cm^−1^; δ_H_ (500 MHz, DMSO-*d*_6_) 7.30 (2H, t, *J* = 9.0 Hz, 3″,5″-H), 7.47–7.47 (3H, m, Ph), 7.72 (2H, t, *J* = 9.0 Hz, 2″,6″-H), 7.87 (1H, d, *J*_trans_ = 16.0 Hz, H_b_), 7.85–7.91 (2H, m, Ph), 8.08 (1H, d, *J* = 9.0 Hz, 7-H), 8.16 (1H, dd, *J* = 1.8 Hz and 9.0 Hz, 8-H), 8.04 (1H, d, *J*_trans_ = 16.5 Hz, H_a_), 8.65 (1H, d, *J* = 1.8 Hz, 10-H); ^13^C-NMR (125 MHz, DMSO-*d*_6_) 88.4, 91.9, 115.5, 115.6 (d, ^2^*J*_CF_ = 21.9 Hz), 115.7, 118.6 (d, ^4^*J*_CF_ = 2.9 Hz), 121.7, 129.0, 129.4, 129.7 (2 × C), 131.3, 134.6 (d, ^3^*J*_CF_ = 8.5 Hz), 135.0, 136.4, 142.9, 143.1, 143.7, 149.0, 163.19 (d, ^1^*J*_CF_ = 245.7 Hz); ^19^F-NMR (282 MHz, DMSO-*d*_6_) −113.16 (1F, d, *J* = 4.7 Hz); HRMS (ES): MH^+^, found: 392.1304. C_24_H_15_N_5_F^+^ requires: 392.1311. Anal. calcd. for C_16_H_14_N_5_F: C, 73.65; H, 3.61; N, 17.89. Found: C, 73.59; H, 3.60; N, 17.64.

*(E)-9-((4-Fluorophenyl)ethynyl)-5-(4-fluorostyryl)tetrazolo[1,5-c]quinazoline* (**5b**). Solid (0.12 g, 48%), *R_f_* 0.54, m.p. 204–206 °C; ν_max_ (ATR) 508, 812, 834, 1234, 1506, 1588, 1635, 2361, 3023 cm^−1^; δ_H_ (300 MHz, DMSO-*d*_6_) 7.33 (2H, t, *J* = 9.0 Hz, 3′,5′-H), 7.33 (2H, t, *J* = 9.0 Hz, 3″,5″-H), 7.73 (2H, t, *J* = 9.0 Hz, 2′,6′-H), 7.91 (1H, d, *J*_trans_ = 16.0 Hz, H_b_), 8.04 (2H, t, *J* = 9.0 Hz, 2″,6″-H), 8.12 (1H, dd, *J =* 1.2 and 8.4 Hz, 8-H), 8.12 (1H, d, *J =* 8.4 Hz, 7-H), 8.39 (1H, d, *J*_trans_ = 16.0 Hz, H_a_), 8.67 (1H, d, *J* = 1.2 Hz, 10-H); ^13^C-NMR (75 MHz, DMSO-*d*_6_) 88.8, 90.4, 116.3 (d, ^2^*J*_CF_ = 21.8 Hz), 116.5 (d, ^2^*J*_CF_ = 21.8 Hz), 118.8 (d, ^4^*J*_CF_ = 3.0 Hz), 120.9, 126.9, 128.4, 129.1, 129.3, 129.6 (d, ^4^*J*_CF_ = 2.8 Hz), 129.9, 134.1, 134.4 (d, ^3^*J*_CF_ = 8.5 Hz), 134.5 (d, ^3^*J*_CF_ = 8.5 Hz), 136.6, 137.4, 151.2, 161.4 d, ^1^*J*_CF_ = 245.7 Hz), 163.1 (d, ^1^*J*_CF_ = 244.7 Hz); ^19^F-NMR (282 MHz, DMSO-*d*_6_) −109.2 (1F, ddd, *J* = 6.6, 9.9 and 20.2 Hz), −109.5 (1F, ddd, *J* = 3.3, 8.5, 16.5 Hz); HRMS (ES): MH^+^, found: 410.1216. C_24_H_14_N_5_F_2_^+^ requires: 410.1217. Anal. calcd. for C_16_H_13_N_5_F_2_: C, 70.41; H, 3.20; N, 17.11. Found: C, 70.39; H, 2.99; N, 17.01.

*(E)-5-(4-Chlorostyryl)-9-((4-fluorophenyl)ethynyl)tetrazolo[1,5-c]quinazoline* (**5c**). Solid (0.16 g, 52%), *R_f_* 0.61, m.p. 235–237 °C; ν_max_ (ATR) 520, 820, 1091, 1232, 1495, 1587, 1633, 2216, 3059 cm^−1^; δ_H_ (500 MHz, DMSO-*d*_6_) 7.35 (2H, t, *J* 8.7 Hz, 3″,5″-H), 7.52 (2H, d, *J* = 8.1 Hz, 3′,5′-H), 7.89 (1H, d, *J*_trans_ = 16.0 Hz, H_b_), 7.92 (2H, d, *J* = 8.1 Hz, 2′,6′-H), 7.93 (2H, t, *J* 8.7 Hz, 2″,6″-H), 8.13 (1H, d, *J* = 8.1 Hz, 7-H), 8.30 (1H, d, *J*_trans_ = 16.0 Hz, H_a_), 8.28 (1H, dd, *J* = 1.2 Hz and 9.0 Hz, 8-H), 8.67 (1H, d, *J* = 1.2 Hz, 10-H); ^13^C-NMR (125 MHz, DMSO-*d*_6_) 88.4, 90.4, 115.5, 115.6 (d, ^2^*J*_CF_ = 21.9 Hz), 115.7, 118.6 (d, ^4^*J*_CF_ = 2.9 Hz), 123.1, 127.1, 129.1, 129.2, 129.7, 131.3, 134.6 (d, ^3^*J*_CF_ = 8.5 Hz), 135.0, 136.4, 142.9, 143.1, 143.7, 149.0, 162.9 (d, ^1^*J*_CF_ = 247.5 Hz); ^19^F-NMR (282 MHz, DMSO-*d*_6_) −113.70 (1F, dd, *J* = 4.7 and 14.6 Hz); HRMS (ES): MH^+^, found: 426.0916. C_24_H_14_N_5_^35^ClF^+^ requires: 426.0922. Anal. calcd. for C_16_H_13_N_5_FCl: C, 67.69; H, 3.08; N, 16.45. Found: C, 67.70; H, 3.06; N, 16.41.

### 3.6. Typical Procedure for the One-Pot S_N_Ar-Heteroannulation and Sonogashira Cross-Coupling of ***2a**–**c*** to Afford ***5a**–**c***

A mixture of **2a** (0.40 g, 1.16 mmol) and sodium azide (0.08 g, 1.24 mmol) in DMF ( 20 mL) was stirred at 45 °C for 4 h. 4-Fluorophenylacetylene (0.21 g, 1.71 mmol), PdCl_2_(P(Cy)_3_)_2_ (0.04 g, 0.06 mmol), CuI (0.01 g, 0.06 mmol), Cs_2_CO_3_ (0.45 g, 1.37 mmol) and water (5 mL) were added to the mixture under nitrogen atmosphere. The mixture was heated at 90 °C for 4 h and then poured into ice-cold water. The product was extracted into chloroform and the combined organic phases were washed with brine, dried over MgSO_4_ and the salt was filtered off. The solvent was evaporated under reduced pressure and the residue was purified by column chromatography on silica gel. Compounds **5a**–**c** were prepared in this fashion; see Table 2 under Scheme 3 for the corresponding yields.

### 3.7. Cytotoxicity Screening Protocol

The human tumour cell lines, HeLa and MCF7, were grown in RPMI and DMEM respectively and supplemented with 10% foetal bovine serum and 2 mM l-glutamine. For a screening experiment, cells were inoculated into 96 well microtiter plates at a density of 6000 cells/well using a volume of 100 μL in each well. After cell inoculation, the microtiter plates were incubated at 37 °C, 5% CO_2_, and 100% relative humidity for approximately 24 h prior to addition of test compounds. At the time of test compound addition, the samples were diluted to double the desired final maximum test concentration with complete medium and additional dilutions were made to provide five drug concentrations. Aliquots of 100 µL of these different drug dilutions were added to the appropriate microtiter wells already containing 100 µL of medium, resulting in the required final drug concentrations. Following test sample addition, the plates were incubated for a further 48 h at 37 °C, 5% CO_2_, in a humidified incubator. At the end of this incubation period the medium was removed and replaced with fresh culture medium containing MTT at a final concentration of 0.5 mg/mL. The 96-well plates were returned to the incubator and incubated for an additional 3 h following which the medium was removed, the MTT crystals solubilized in DMSO and absorbance measured at 560 nm using a BioTek PowerWave XS Spectrophotometer (BioTech Instrument, Winooski, VT, USA).

### 3.8. Methodology for Docking Studies

Molecular docking of compounds **3a**, **3b** and **4e** to the 3D structure of a tubulin heterodimer (PDB code:1TUB [15] was carried out using the CDOCKER protocol [16] in Discovery Studio 2017. Prior to performing the docking, compounds were drawn using Discovery Studio and prepared using the ‘Prepare Ligand’ protocol. The protein structure was downloaded from the Protein Data Bank, prepared using the ‘Prepare Protein’ protocol in Discovery Studio which included removing any existing ligands bound to the model and binding sites defined from receptor cavities. The receptor cavity in this tubulin structure is present between the two monomers of the tubulin dimer structure. Docking was performed using default settings and the best conformation of the ligand selected and evaluated.

## 4. Conclusions

In summary, we have demonstrated that the 6-bromo-4-chloro-2-styrylquinazoline scaffold undergoes sequential nucleophilic aromatic substitution-heteroannulation and palladium catalyzed Suzuki-Miyaura or Sonogashira cross-coupling reactions to afford novel 9-carbo substituted 5-styryltetrazolo[1,5-*c*]quinazolines in a single-pot operation. The tetrazoloquinazolines evaluated for anti-proliferative activity in this investigation were found to exhibit varying degrees of toxicity towards MCF-7 and HeLa cells (see Supplementary Materials S2, S3). Complete loss of cytotoxicity was observed for derivatives substituted with a 4-fluorophenyl ring directly or via a π-conjugated bridge (C=C or C≡C bond) except for the 9-(4-fluorostyryl) derivative **4e**, which was found to exhibit selectivity and significant cytotoxicity against the MCF-7 cell line. It can be concluded that the presence of a bulky group at the 9-position of the tetrazoloquinazolines is undesirable for cytotoxicity. Docking studies revealed that the tetrazoloquinazolines that showed anti-proliferative activity could act by binding tubulin at the dimer interface. The binding of the compounds to tubulin would prevent tubulin polymerization or disassociation and presumably result in apoptosis.

## Figures and Tables

**Table 1:** Percentage yields of **4a**–**f**.

Compound	Ar^1^	Ar^2^	%Yield from 3	%Yield from 2
**4a**	C_6_H_5_-	4-FC_6_H_4_-	53	65
**4b**	4-FC_6_H_4_-	4-FC_6_H_4_-	51	60
**4c**	4-ClC_6_H_4_-	4-FC_6_H_4_-	50	63
**4d**	C_6_H_5_-	4-FC_6_H_4_CH=CH-	47	57
**4e**	4-FC_6_H_4_-	4-FC_6_H_4_CH=CH-	58	62
**4f**	4-ClC_6_H_4_-	4-FC_6_H_4_CH=CH-	55	68

**Table 2:** Percentage yields of **5a**–**c**.

Compound	Ar	%Yield from 3	%Yield from 2
**5a**	C_6_H_5_-	50	58
**5b**	4-FC_6_H_4_-	48	54
**5c**	4-ClC_6_H_4_-	52	55

**Table 3:**
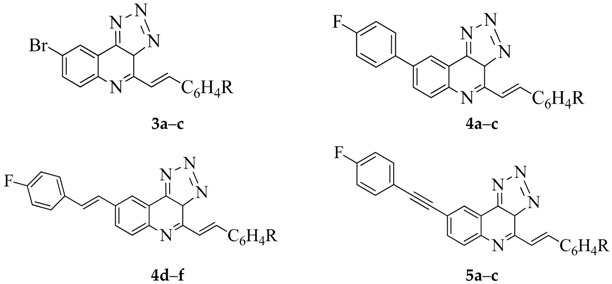
Cytotoxic effects of **3a**–**c**, **4a**–**f** and **5a**–**c** against MCF-7 and HeLa cell lines.

Compound	R	IC_50_ Values	
		MCF-7	HeLa
**3a**	H	9.6	7.3
**3b**	4-F	21.0	22.4
**3c**	4-Cl	88.6	121.0
**4a**	H	>200	>200
**4b**	4-F	85.9	196.0
**4c**	4-Cl	>200	>200
**4d**	H	>200	>200
**4e**	4-F	62.0	184.0
**4f**	4-Cl	>200	>200
**5a**	H	>200	>200
**5b**	4-F	>200	>200
**5c**	4-Cl	>200	>200
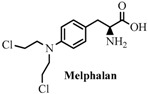		61.0	88.9

## Supplementary Materials

The NMR spectra of compounds **2**–**5** and the percentage cell viability (±standard deviation) as well as the linear regression plots (used to calculate IC_50_ values) for Melphalan and compounds **3**–**5** are listed in the supplementary materials.

## References

[1] Khan I., Ibrar A., Abbas N., Saeed A. (2014). Recent advances in the structural library of functionalized quinazoline and quinazolinone scaffolds: Synthetic approaches and multifarious applications. Eur. J. Med. Chem..

[2] Romagnoli R., Baraldi P.G., Salvador M.K., Preti D., Tabrizi M.A., Brancale A., Fu X.-H., Li J., Zhang S.-Z., Hamel E. (2012). Synthesis and evaluation of 1,5-disubstituted tetrazoles as rigid analogues of combretastatin A-4 with potent antiproliferative and antitumor activity. J. Med. Chem..

[3] Krishna S.M., Padmalatha Y., Ravindranath L.K. (2015). Tetrazole as a core unit biological evaluation agent. Int. J. Med. Pharm. Res..

[4] Ichinari D., Nagaki A., Yoshida J.-I. (2017). Generation of hazardous methyl azide and its application to the synthesis of a key-intermediate of picarbutrazox, a new potent pesticides in flow. Bioorg. Med. Chem..

[5] Jordan M.A. (2002). Mechanism of action of antitumor drugs that interact with microtubules and tubulin. Curr. Med. Chem. Anti-Cancer Agents.

[6] Shaban M.A.E., Taha M.A.M., Sharshira E.M. (1991). Synthesis and biological activities of condensed heterocyclo[n,m-*a,b*, or *c*]quinazolines. Adv. Heterocycl. Chem..

[7] Agbo E.N., Makhafola T.J., Choong Y.S., Mphahlele M.J., Ramasami P. (2016). Synthesis, biological evaluation and molecular docking studies of 6-aryl-2-styrylquinazolin-4(3*H*)-ones. Molecules.

[8] Purser S., Moore P.R., Swallow S., Gouverneur V. (2008). Fluorine in medicinal chemistry. Chem. Soc. Rev..

[9] Jampilek M., Musiol R., Finster J., Pesko M., Carroll J., Kralova K., Vejsova M., O’Mahony J., Coffey A., Dohnal J. (2010). Investigating biological activity spectrum for novel styrylquinazoline analogues. Molecules.

[10] Antypenko L.M., Kovalenko S.I., Karpenko O.V., Antypenko O.M., Katsev A.M., Achkasova O.M. (2013). Synthesis and hydrolytic cleavage of tetrazolo[1,5-*c*]quinazolines. Sci. Pharm..

[11] Chaurasia M.R., Sharma S.K. (1980). Synthesis of some heterocyclic compounds with bridgehead nitrogen atoms. Heterocycles.

[12] Hagmann W.K. (2008). The many roles of fluorine in medicinal chemistry. J. Med. Chem..

[13] Jiang J.B., Hesson D.P., Dusak B.A., Dexter D.L., Kang G.J., Hamel E. (1990). Synthesis and biological evaluation of 2-styrylquinazolin-4(3*H*)-ones, a new class of antimitotic anticancer agents which inhibit tubulin polymerization. J. Med. Chem..

[14] Chawla P., Batra C. (2013). Recent advances of quinazolinone derivatives as marker for various biological activities. Int. Res. J. Pharm..

[15] Nogales E., Wolf S.G., Downing K.H. (1998). Structure of the alpha beta tubulin dimer by electron crystallography. Nature.

[16] Wu G., Robertson D.H., Brooks C.L., Vieth M.J. (2003). Detailed analysis of grid-based molecular docking: A case study of CDOCKER-A CHARMm-based MD docking algorithm. Comput. Chem..

