# Supplementary material for: Synthesis, Cytotoxicity and Molecular Docking Studies of the 9-Substituted 5-Styryltetrazolo[1,5-c]quinazoline Derivatives
Source: Molecules. 2017 Oct 26;22(11):1719. doi: 10.3390/molecules22111719 (PMC6150304; doi:10.3390/molecules22111719)
Supplement: Supplementary file 1 [file molecules-22-01719-s001.pdf]

Supplementary Information

# Synthesis, Cytotoxicity and Molecular Docking Studies of the 9-Substituted 5-Styryltetrazolo[1,5-*c*]quinazoline Derivatives

**Malose J. Mphahlele [1,*], Samantha Gildenhuys [2] and Nishal Parbhoo [2]**

[1] Department of Chemistry, University of South Africa, Private Bag X06, Florida 1710, South Africa
[2] Department of Life & Consumer Sciences, University of South Africa, Private Bag X06, Florida 1710, South Africa; gildes@unisa.ac.za (S.G.); parbhn1@unisa.ac.za (N.P.)
* Correspondence: mphahmj@unisa.ac.za; Tel.: +27-11-670-6301

**S1.** $^{1}$H-NMR and $^{13}$C-NMR spectra of compounds **2–5**.

**S2.** Cytotoxicity and dose response curves of Melphalan and compounds **3–5** against HeLa cells.

**S3.** Cytotoxicity and dose response curves of Melphalan and compounds **3–5** against MCF-7 cells.

**S4.** Spread sheet for statistical analysis which contains p values for each test.

**S1:** $^{1}$H NMR and $^{13}$C NMR spectra of compounds 2–5

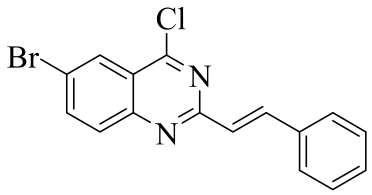


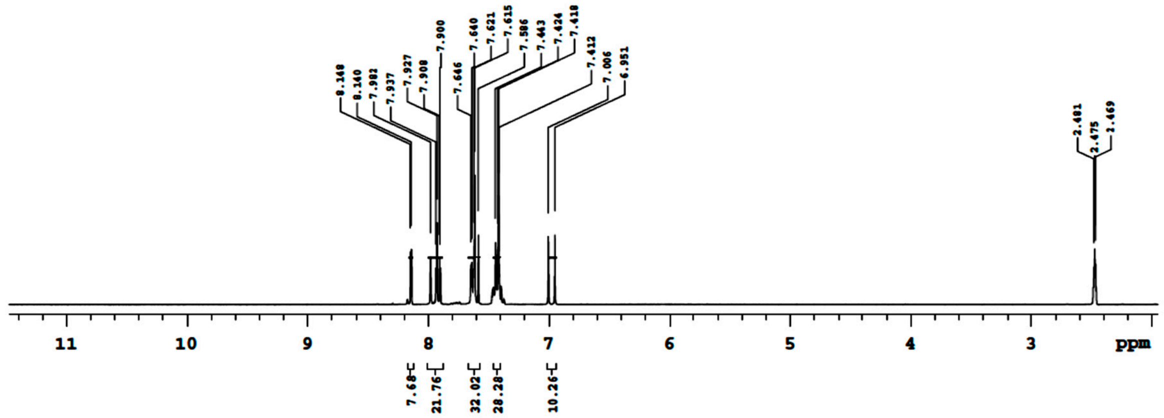


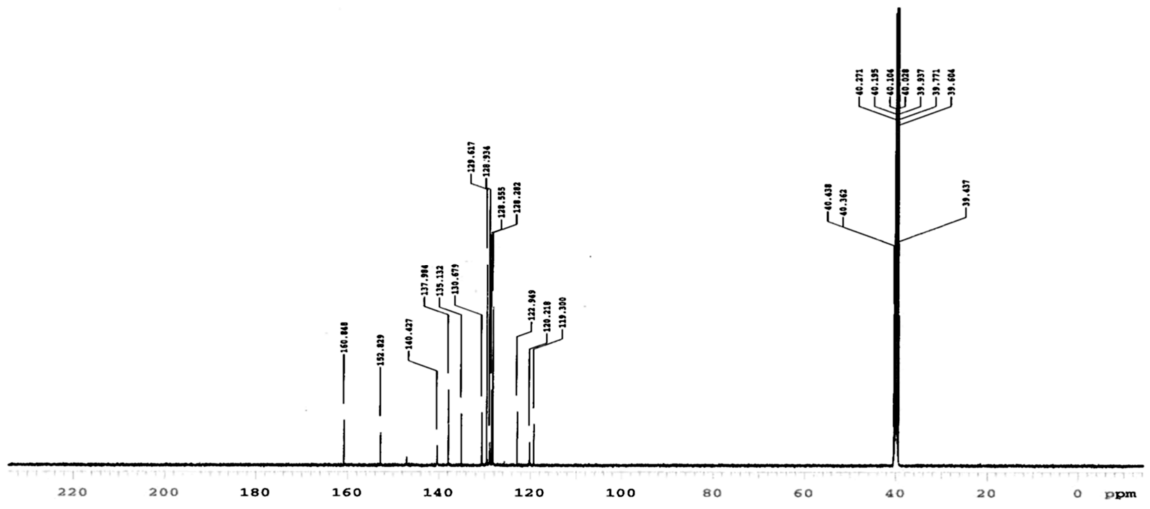


**Figure S-1.1:** $^{1}$H NMR and $^{13}$C NMR spectra of **2a** in DMSO-$d_6$ at 300 MHz and 75 MHz, respectively

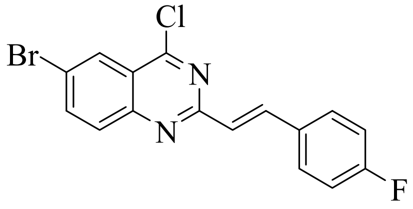


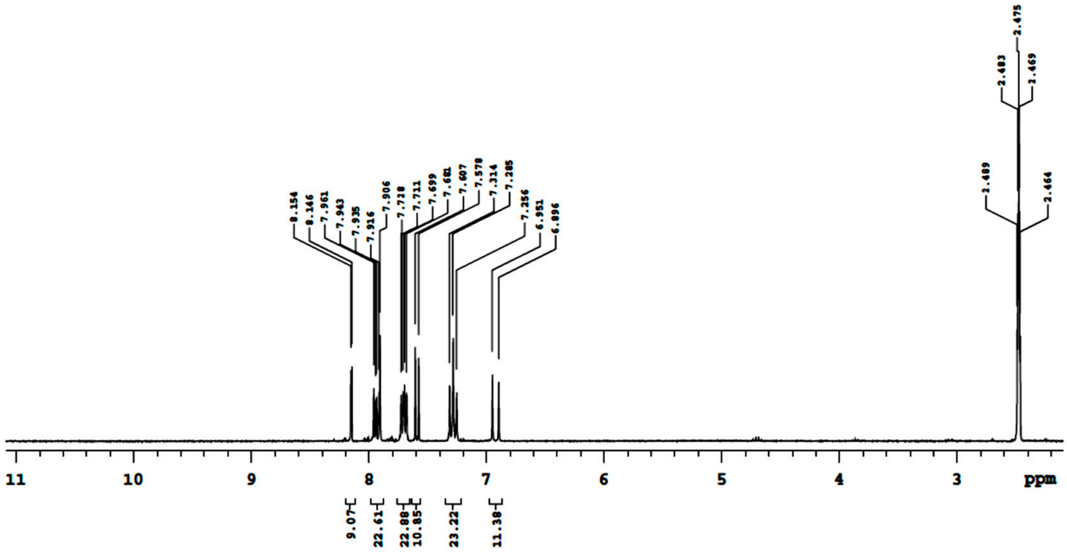


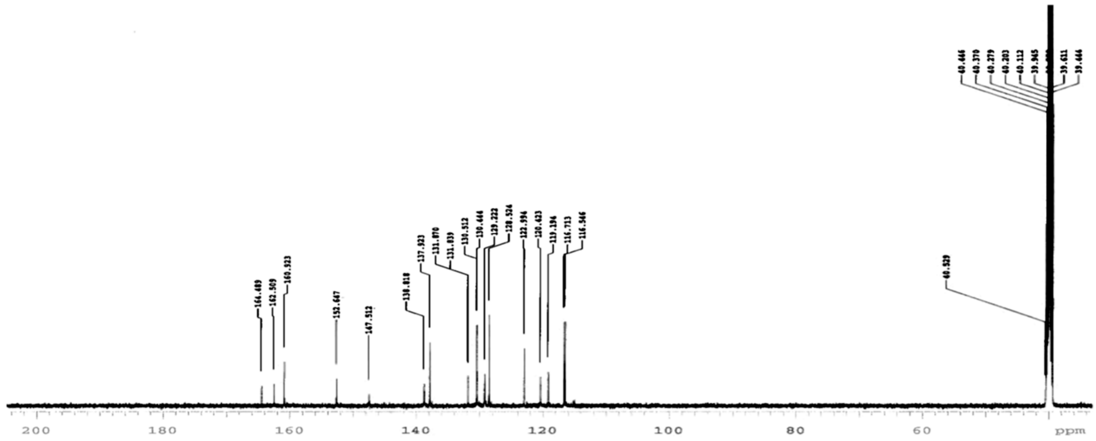


**Figure S-1.2:** $^{1}$H NMR and $^{13}$C NMR spectra of **2b** in DMSO-$d_6$ at 300 MHz and 75 MHz, respectively

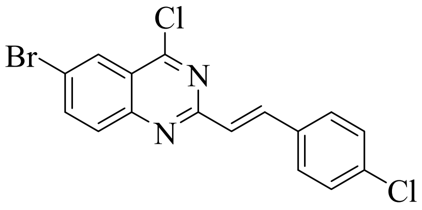


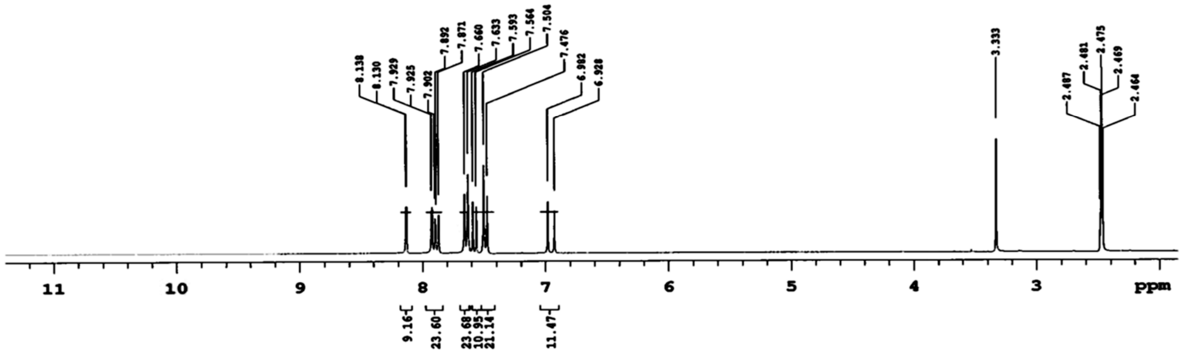

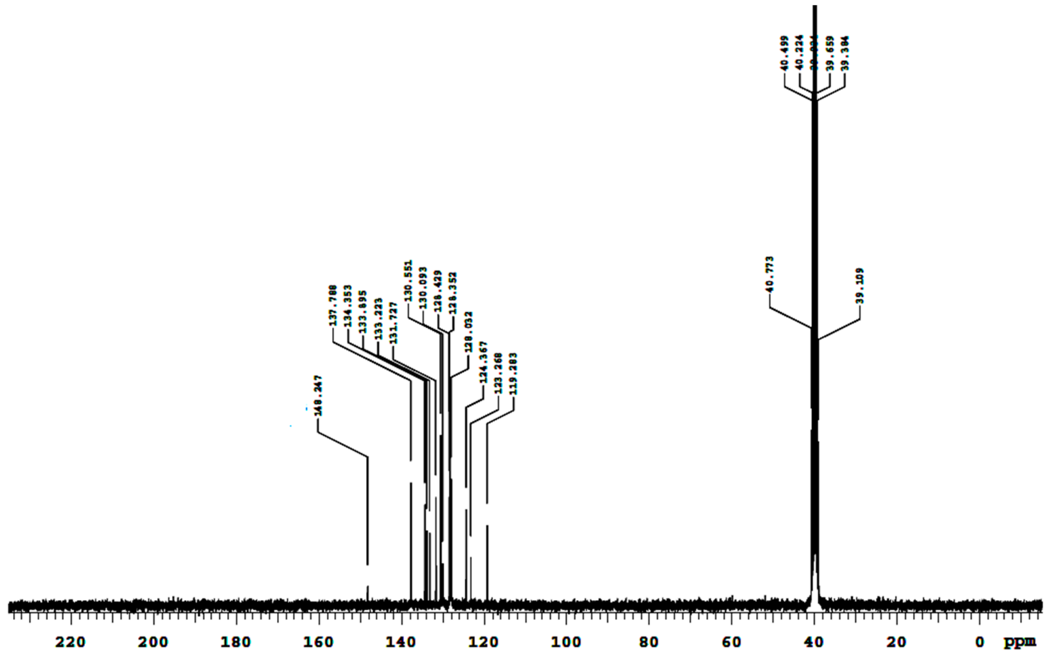

**Figure S-1.3:** $^{1}H$ NMR and $^{13}C$ NMR spectra of **2c** in DMSO-$d_6$ at 300 and 75 MHz, respectively

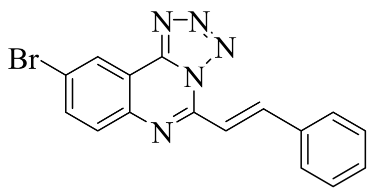

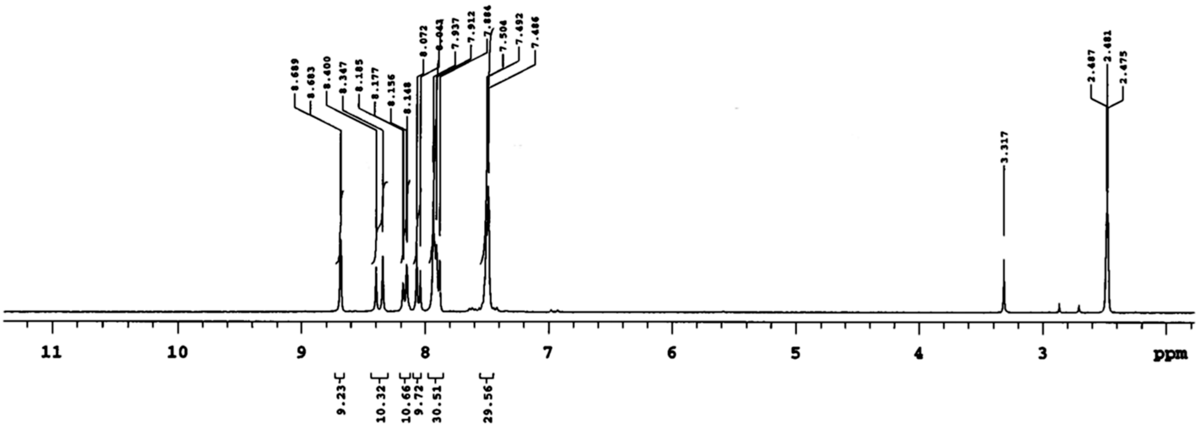

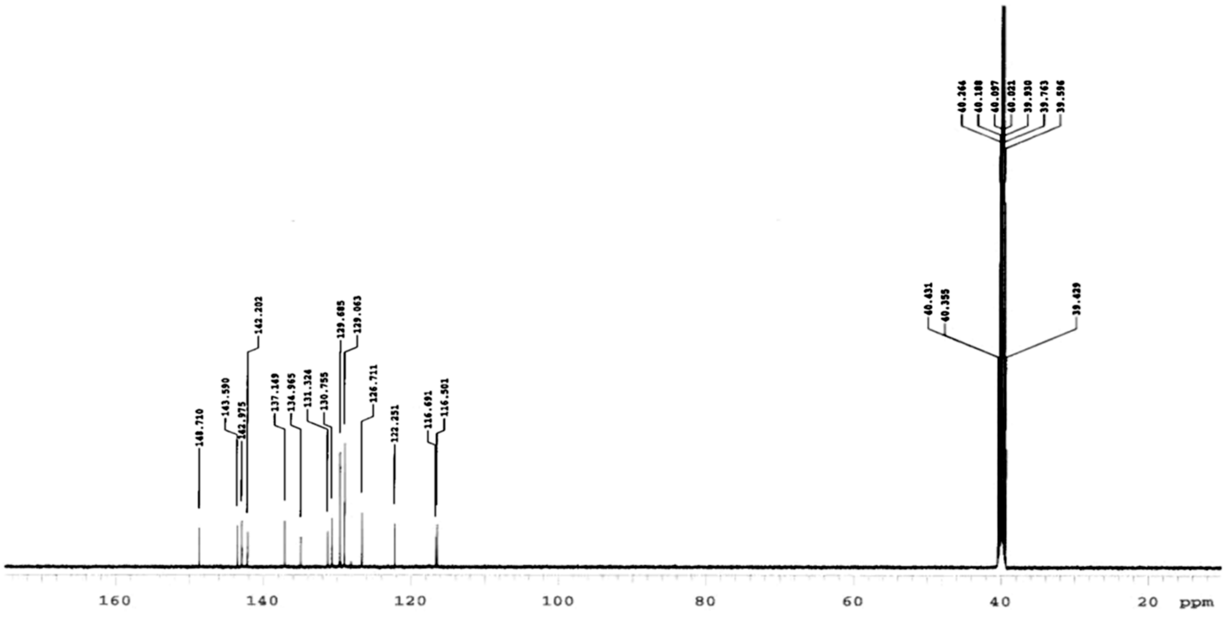

**Figure S-1.4:** $^{1}$H NMR and $^{13}$C NMR spectra of **3a** in DMSO-$d_6$ at 300 MHz and 75 MHz, respectively

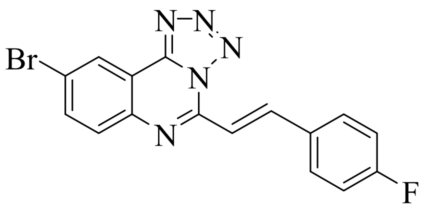


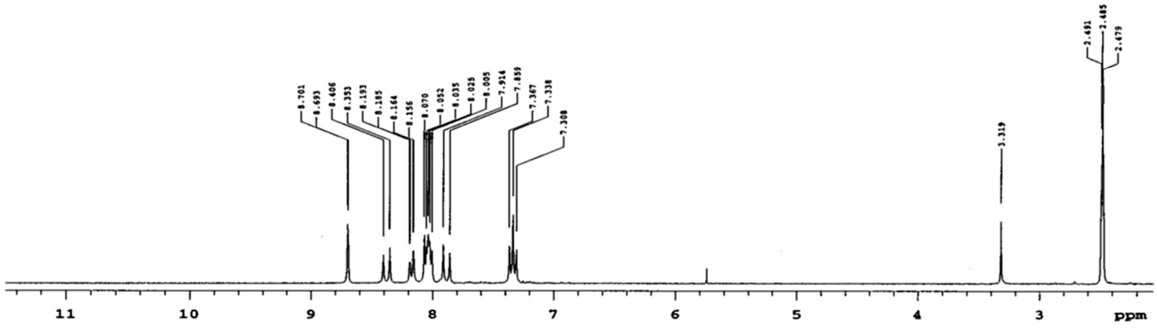


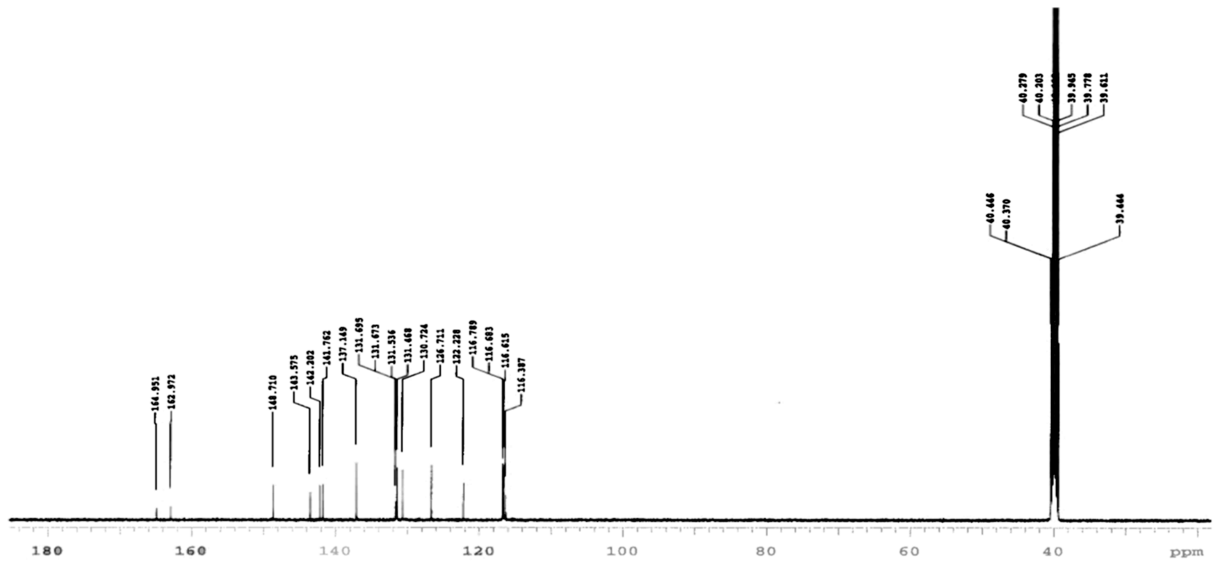


**Figure S-1.5:** $^{1}H$ NMR and $^{13}C$ NMR spectra of **3b** in DMSO-$d_6$ at 300 MHz and 75 MHz, respectively

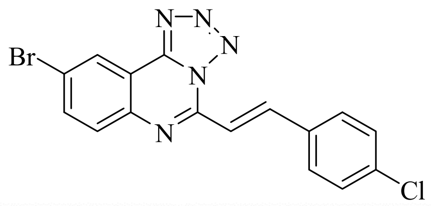


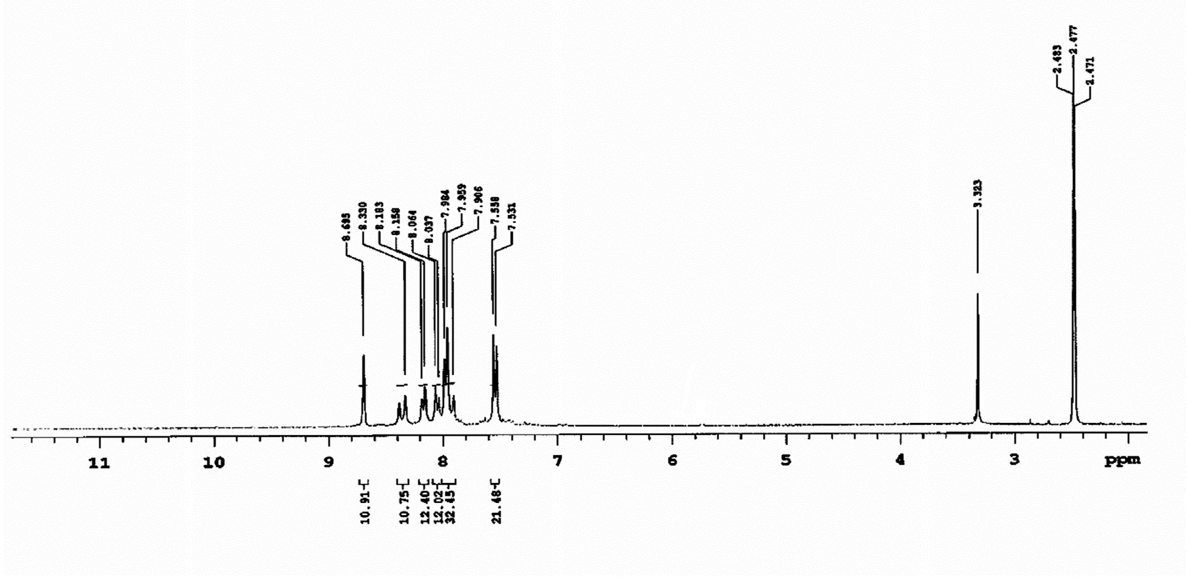


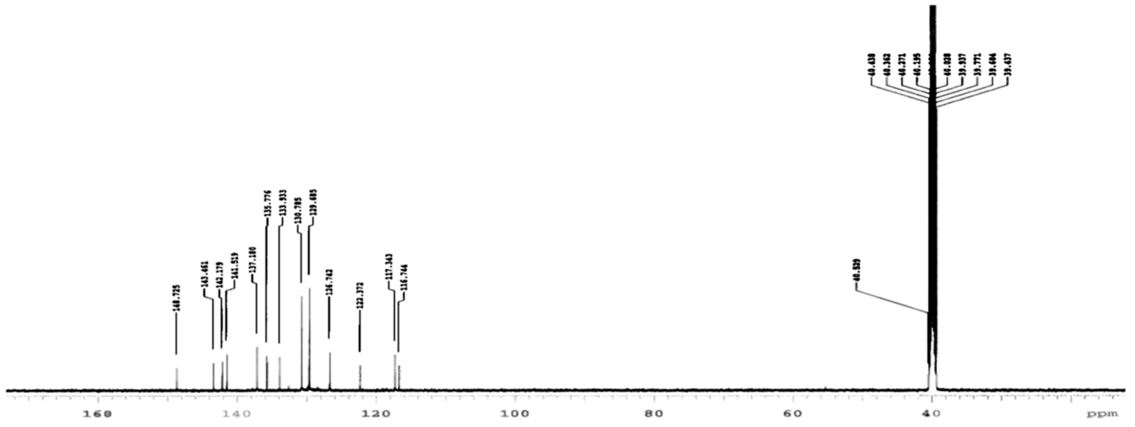


**Figure S-1.6:** $^{1}$H NMR and $^{13}$C NMR spectra of **3c** in DMSO-*d*$_6$ at 300 MHz and 75 MHz, respectively

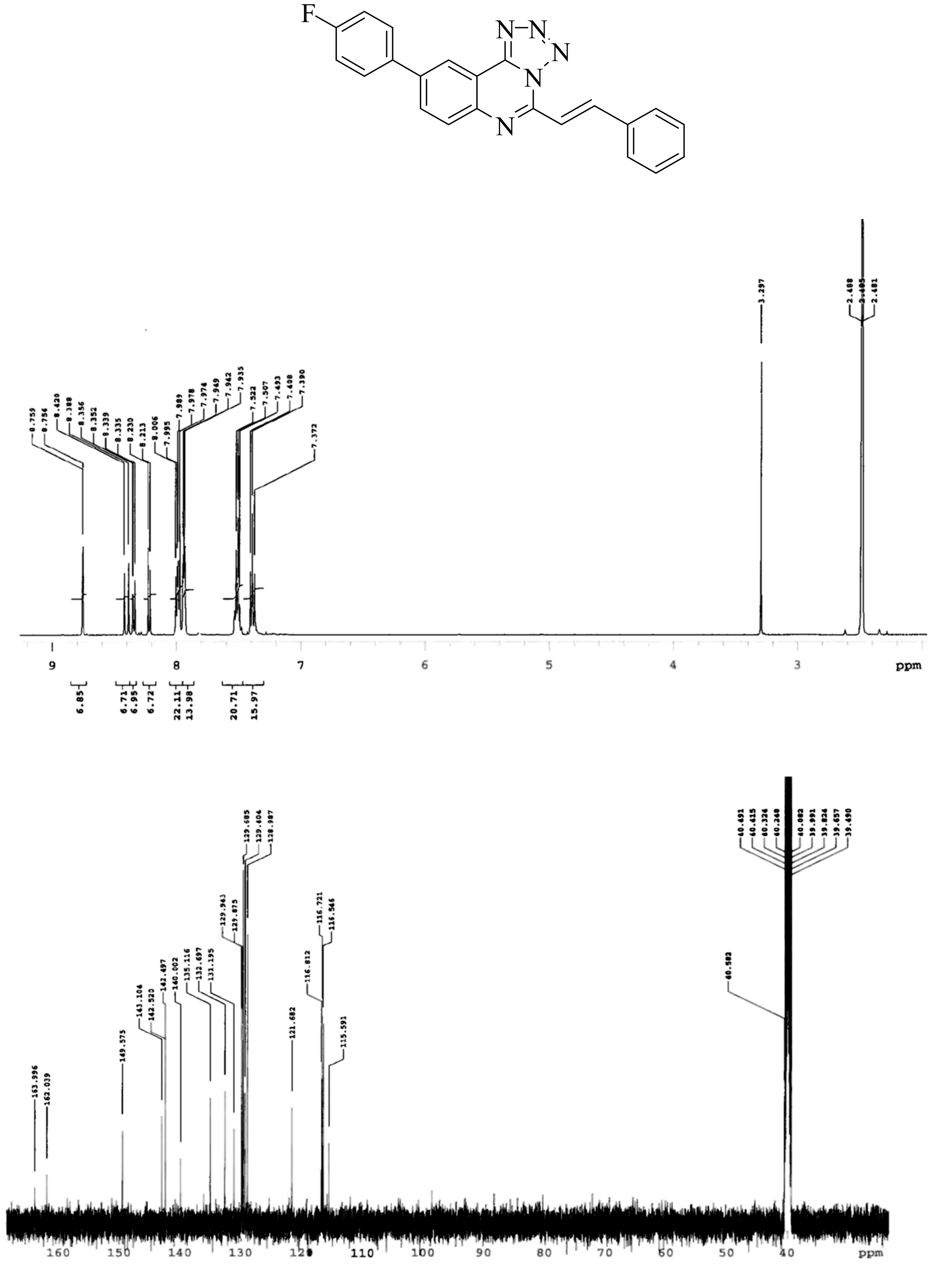


**Figure S-1.7:** $^{1}$H NMR spectrum of **4a** in DMSO-$d_6$ at 500 and 125 MHz, respectively

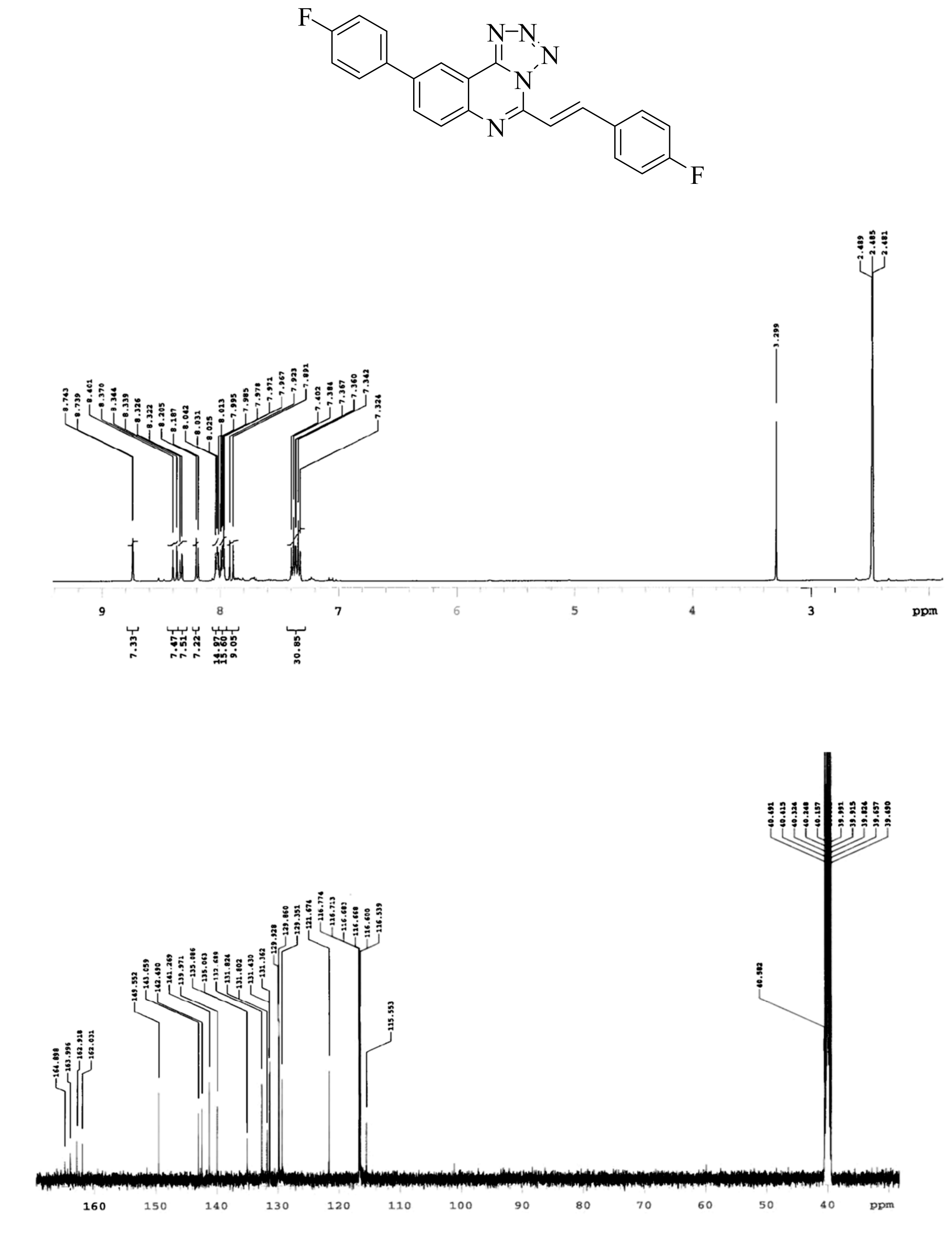


**Figure S-1.8:** $^{1}H$ NMR and $^{13}C$ NMR spectra of **4b** in DMSO-$d_6$ at 500 MHz and 125 MHz, respectively

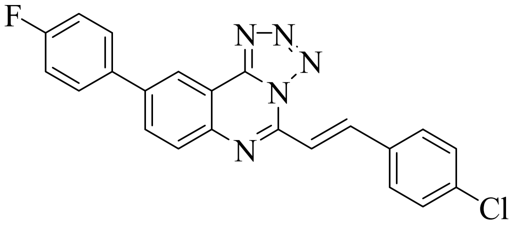

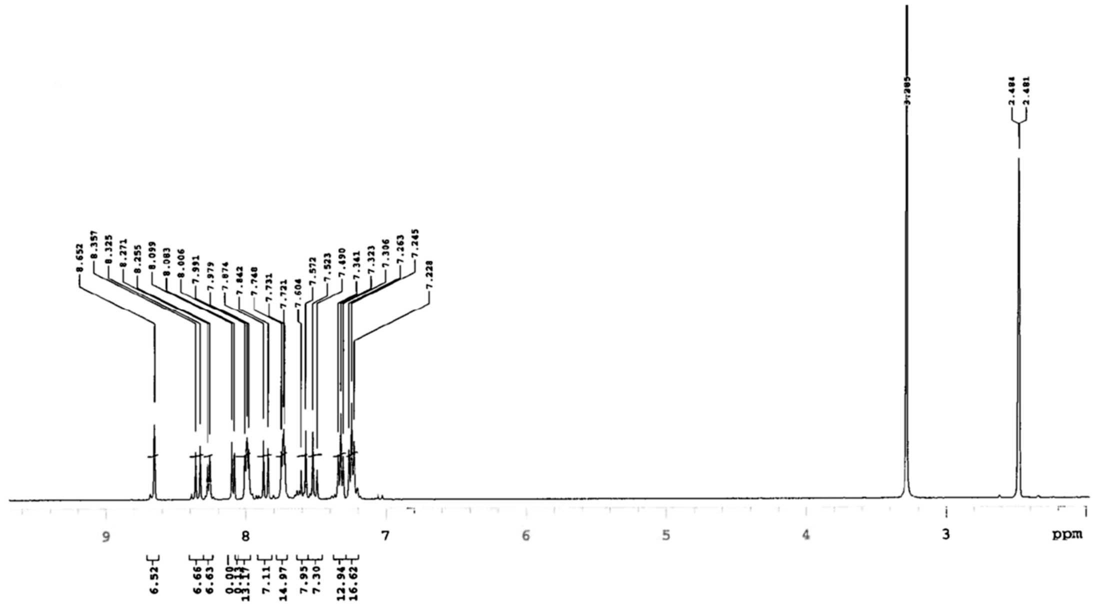

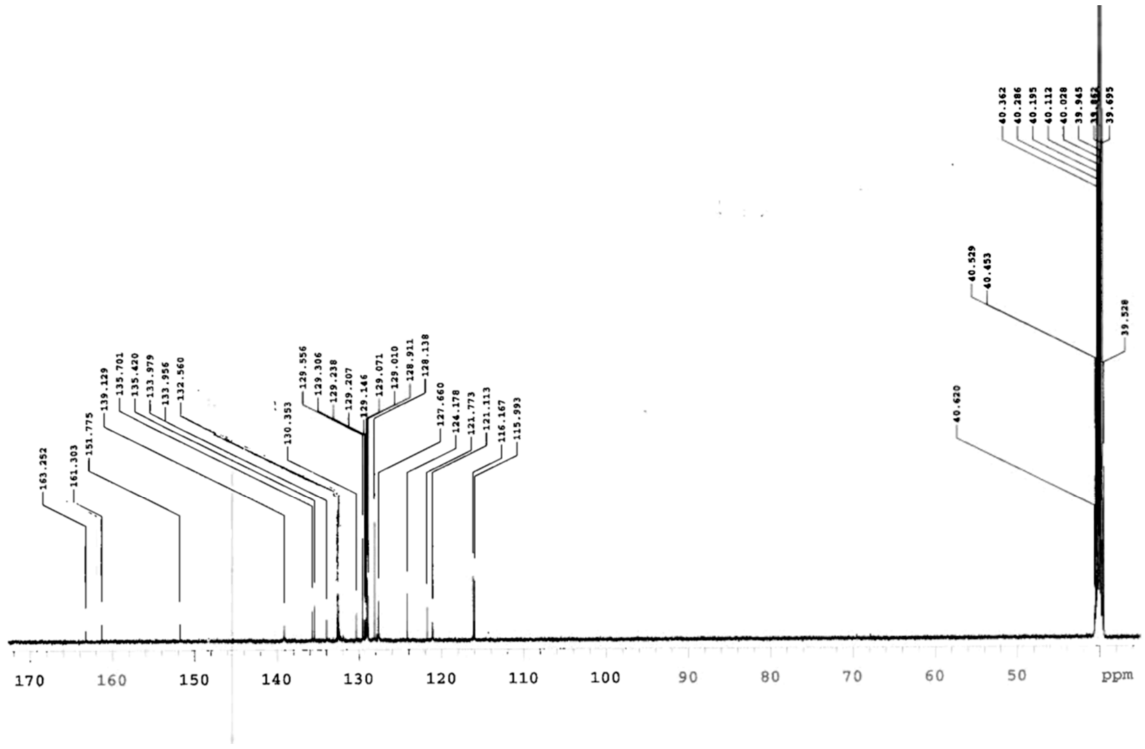

**Figure S-1.9:** $^{1}$H NMR and $^{13}$C NMR spectra of **4c** in DMSO-$d_6$ at 500 and 125 MHz, respectively

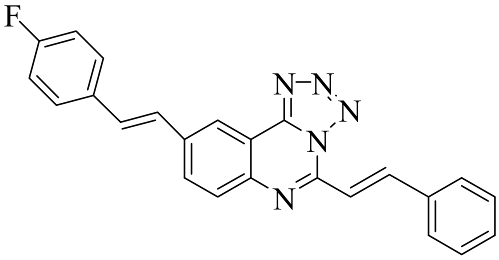

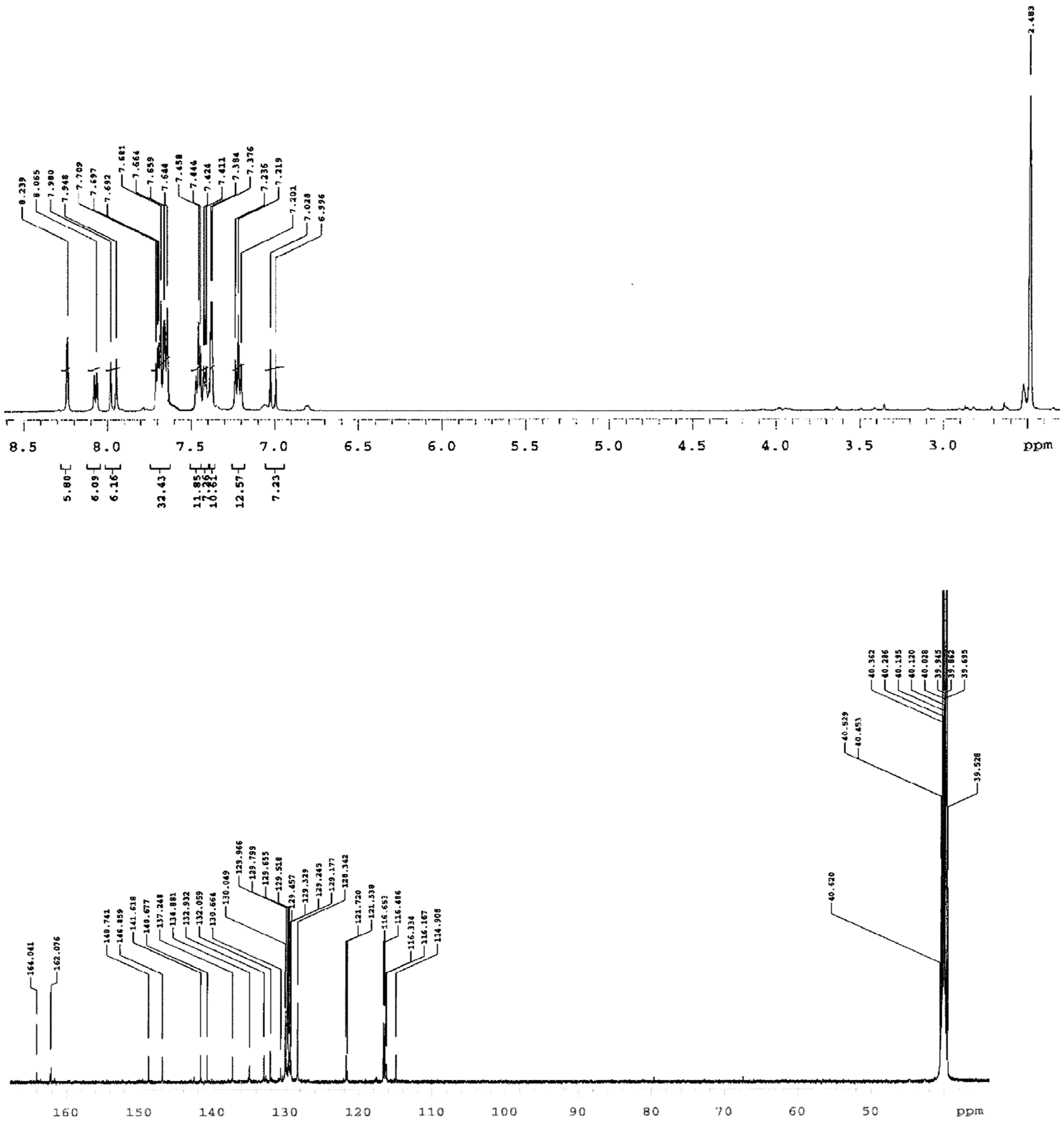

**Figure S-1.10:** $^{1}H$ NMR and $^{13}C$ NMR spectra of **4d** in DMSO-$d_6$ at 500 and 125 MHz, respectively

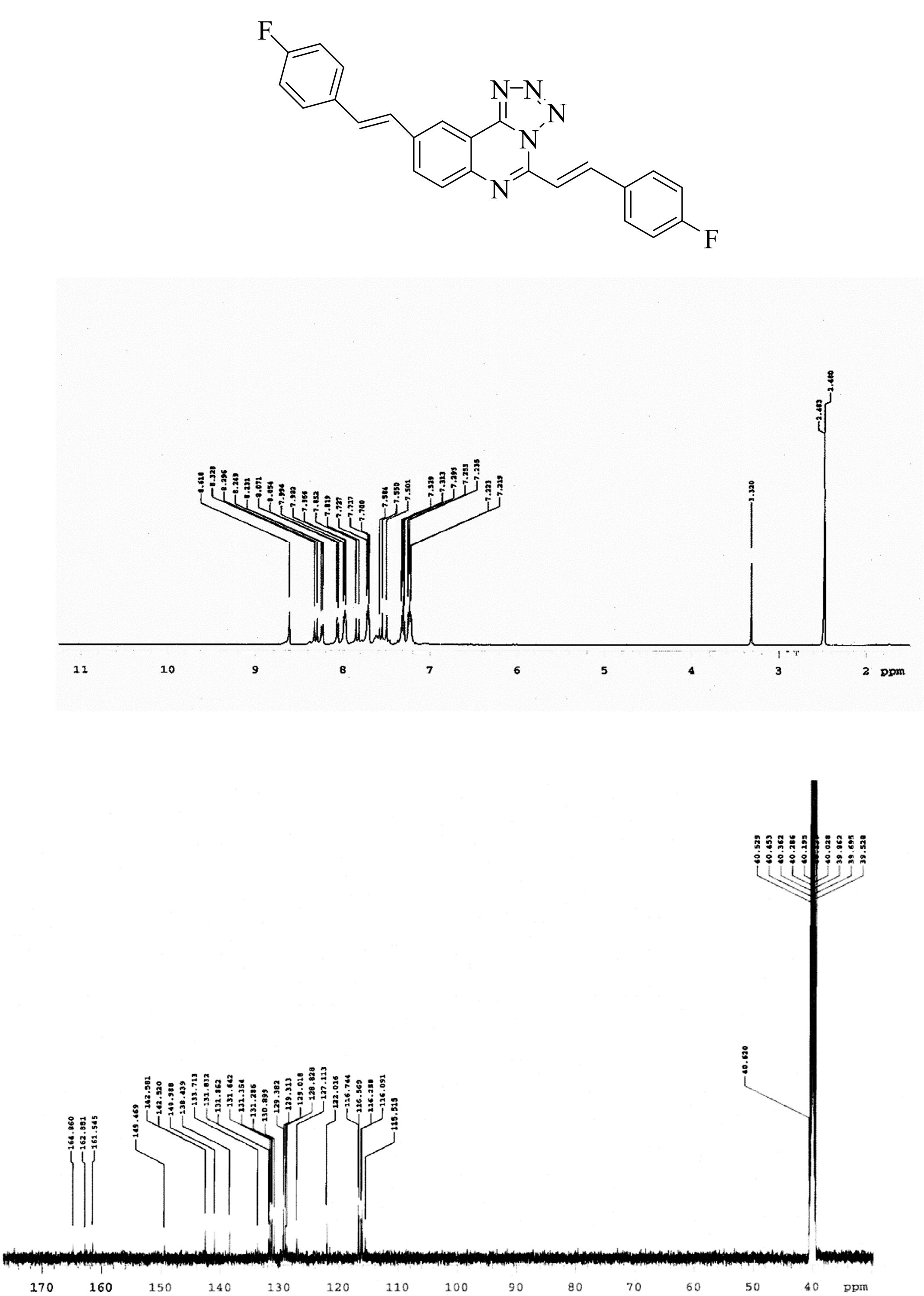

**Figure S-1.11:** $^{1}H$ NMR and $^{13}C$ NMR spectra of **4e** in DMSO-$d_6$ at 300 and 75 MHz, respectively

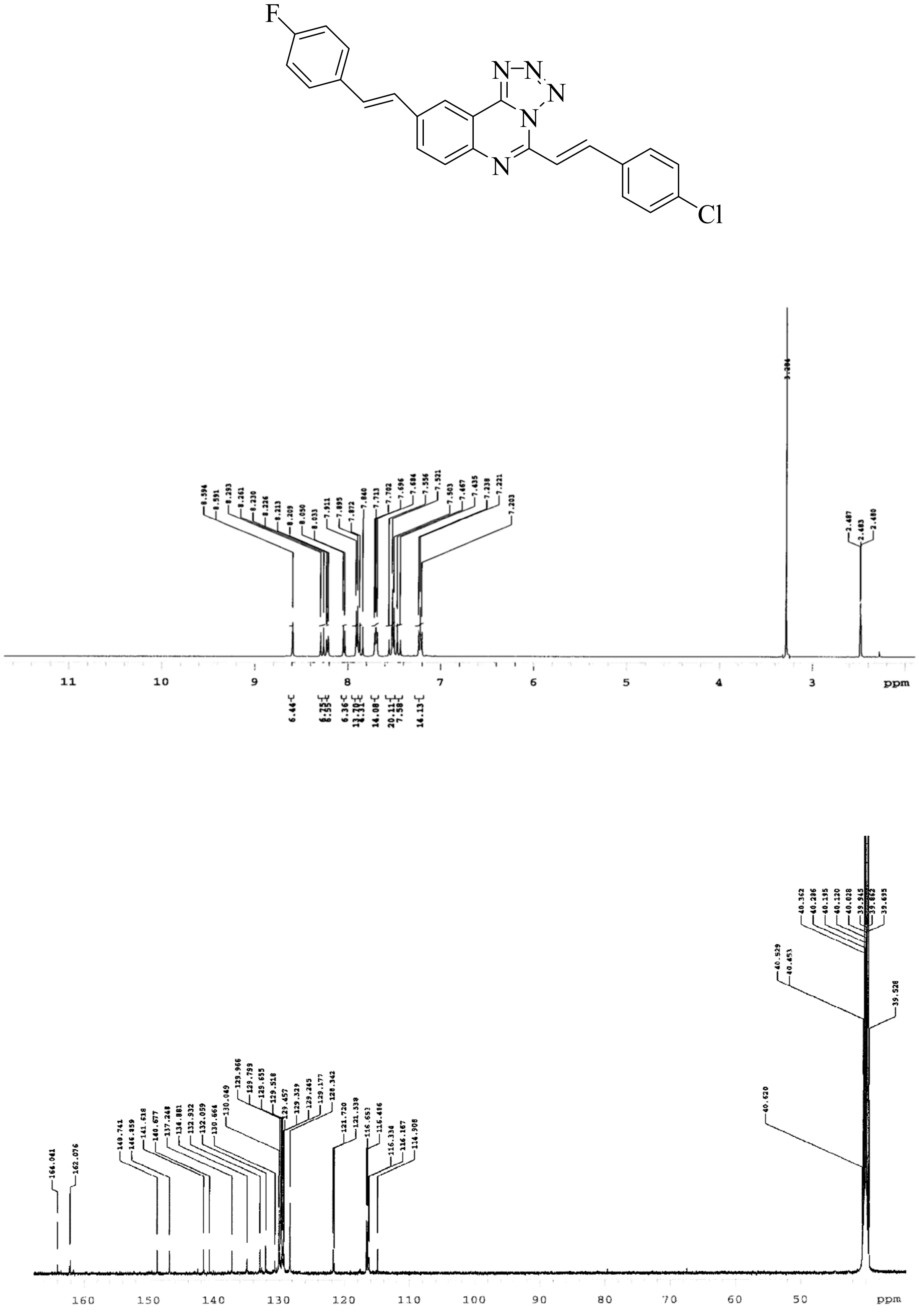


**Figure S-1.12:** $^{1}H$ NMR and $^{13}C$ NMR spectra of **4f** in DMSO-$d_6$ at 500 and 125 MHz, respectively

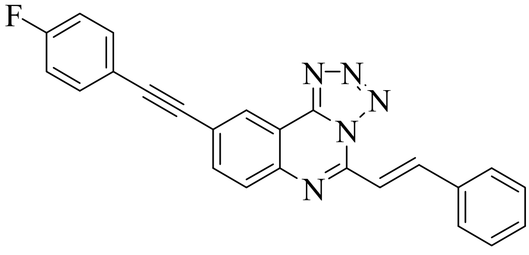

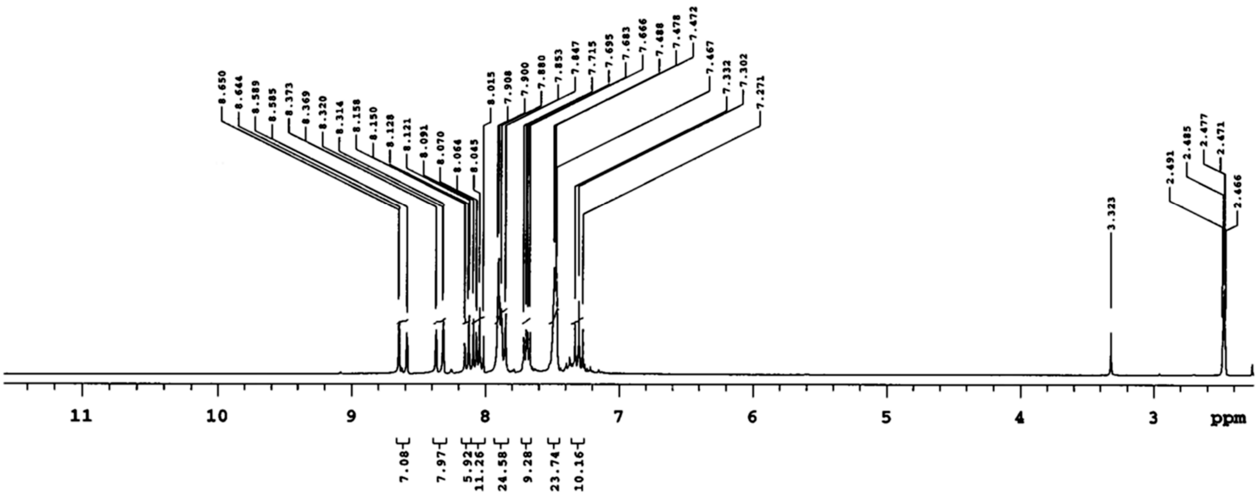


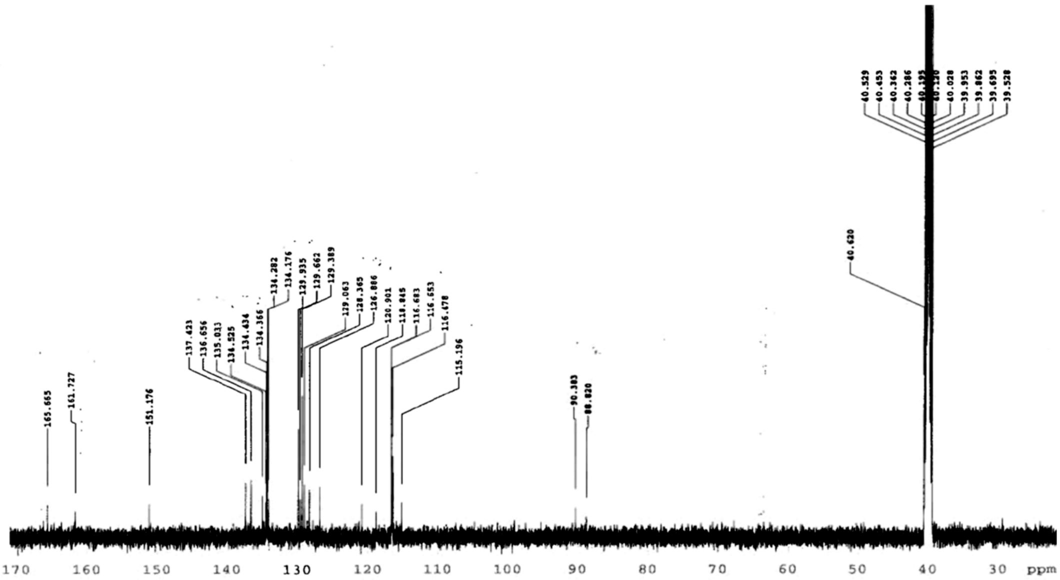


**Figure S-1.13:** $^{1}H$ NMR and $^{13}C$ NMR spectra of **5a** in DMSO-$d_6$ at 500 and 125 MHz, respectively

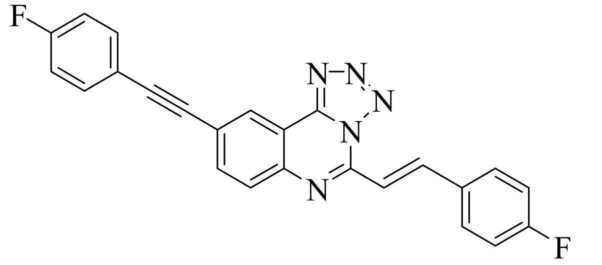

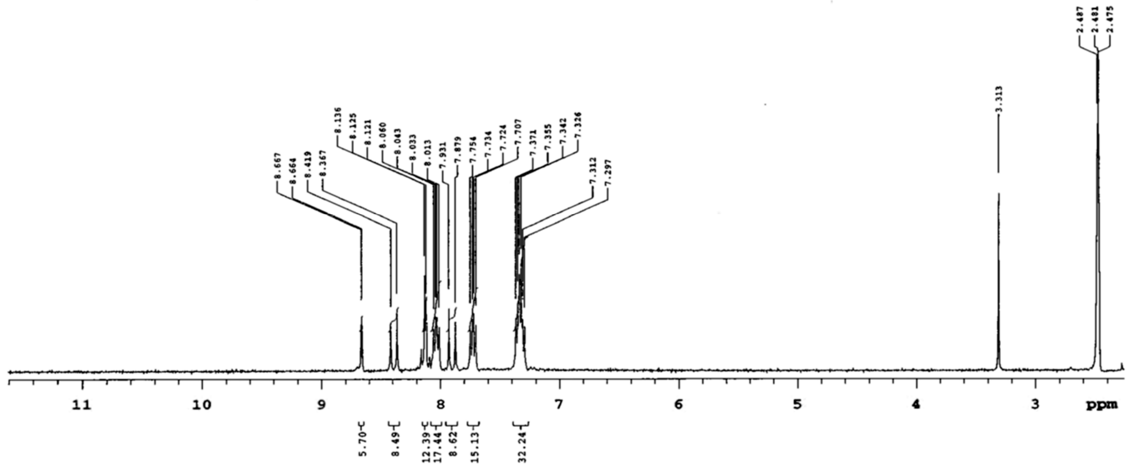


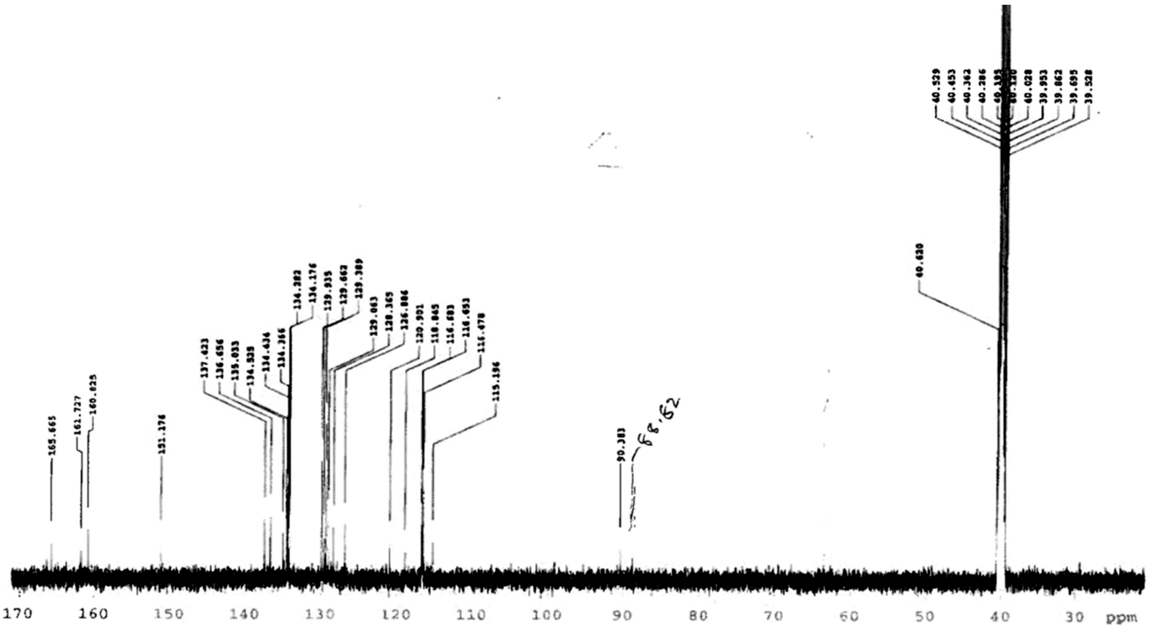


**Figure S-1.14:** $^{1}H$ NMR and $^{13}C$ NMR spectra of **5b** in DMSO-$d_6$ at 300 and 75 MHz, respectively

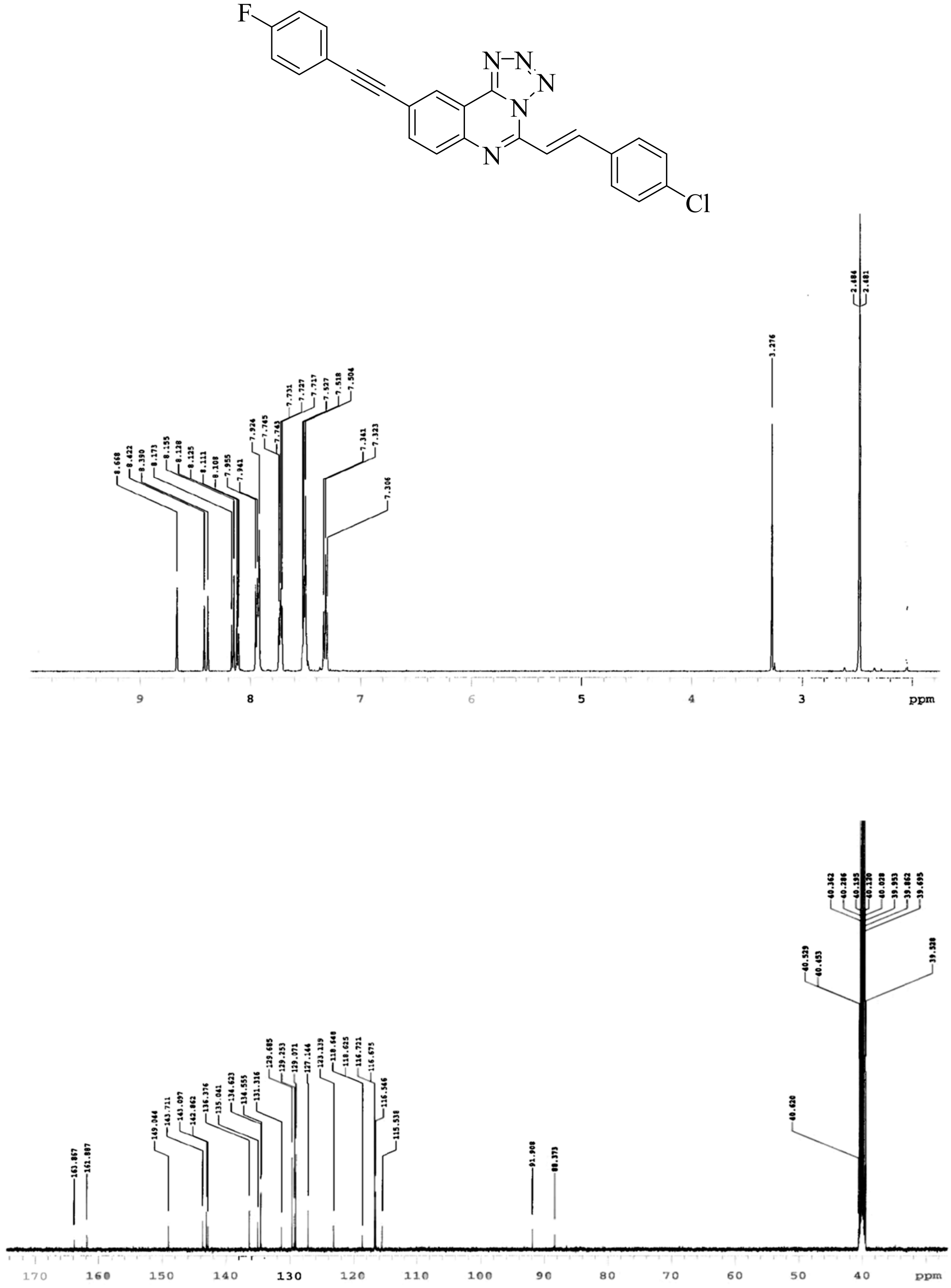

**Figure S-1.15:** $^{1}$H NMR and $^{13}$C NMR spectra of **5c** in DMSO-$d_6$ at 500 and 125 MHz, respectively

**S2: Cytotoxicity and dose response curves of Melphalan and compounds 3–5 against HeLa cells**

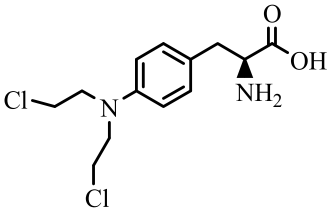


**Melphalan**

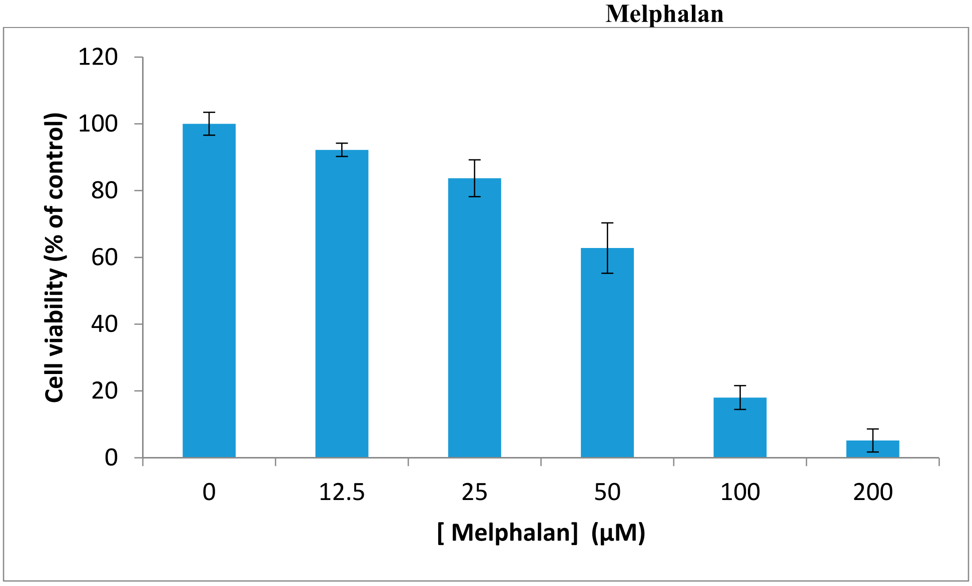


**Figure S-2.1:** Cytotoxicity of **Melphalan** against HeLa cells

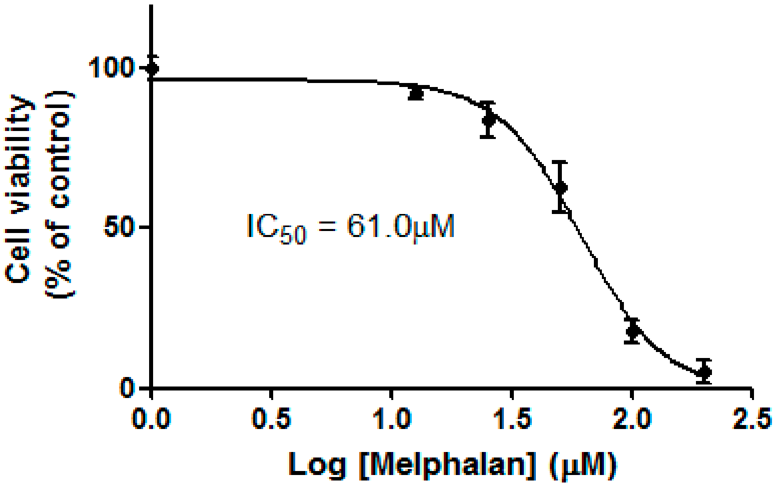


**Figure S-2.2:** Dose response curve of **Melphalan** against HeLa cells

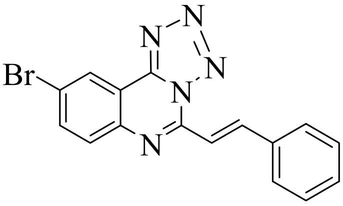


**3a**

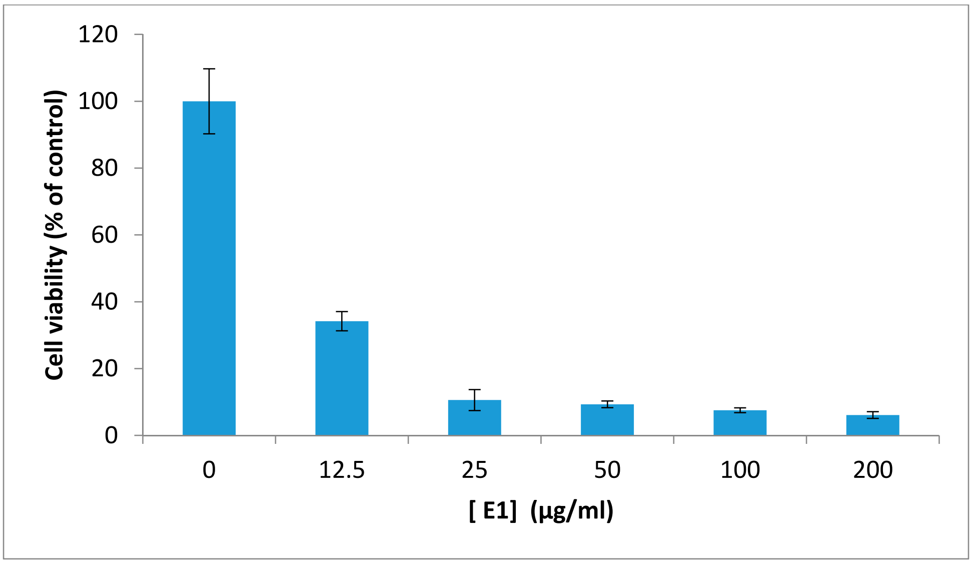


**Figure S-2.3:** Cytotoxicity of **3a** against HeLa cells

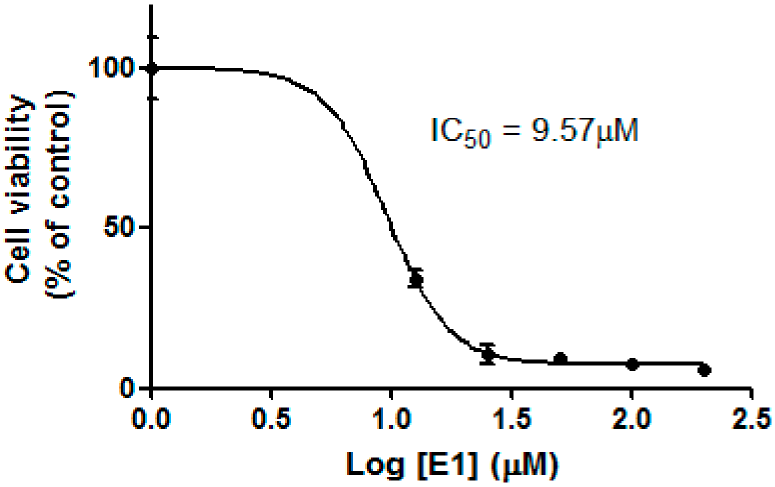


**Figure S-2.4:** Dose response curve of **3a** against HeLa cells

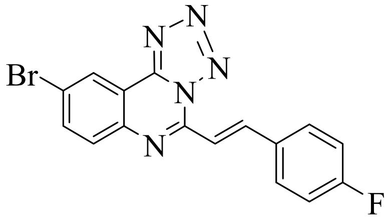


**3b**

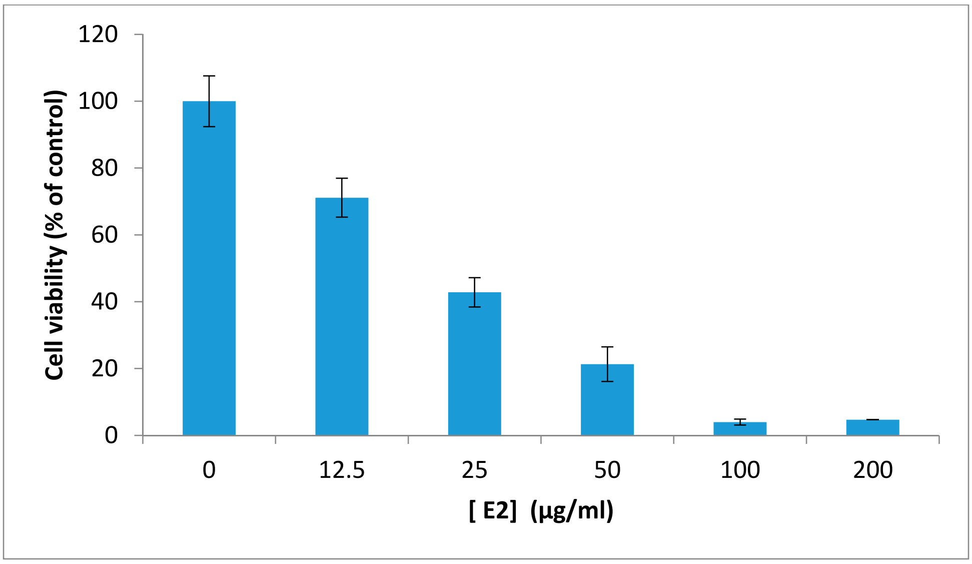


**Figure S-2.5:** Cytotoxicity of **3b** against HeLa cells

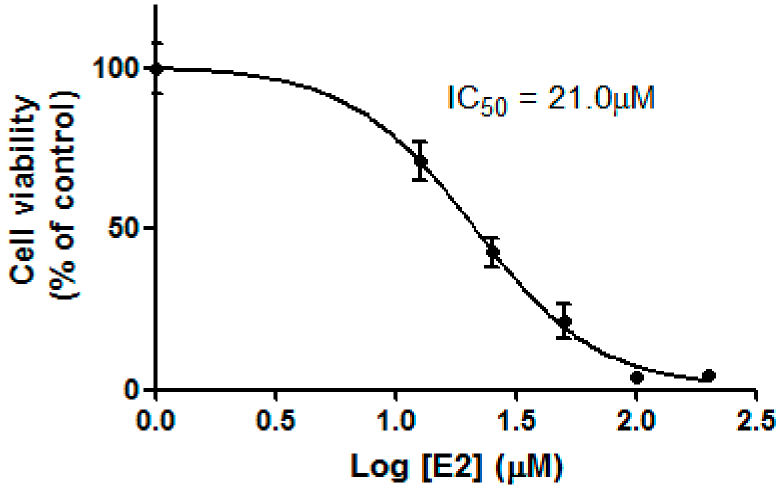


**Figure S-2.6:** Dose response curve of **3b** against HeLa cells

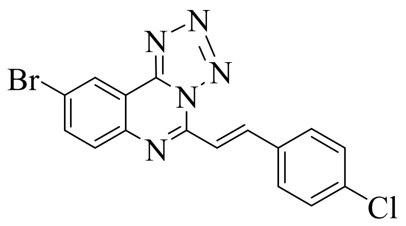


**3c**

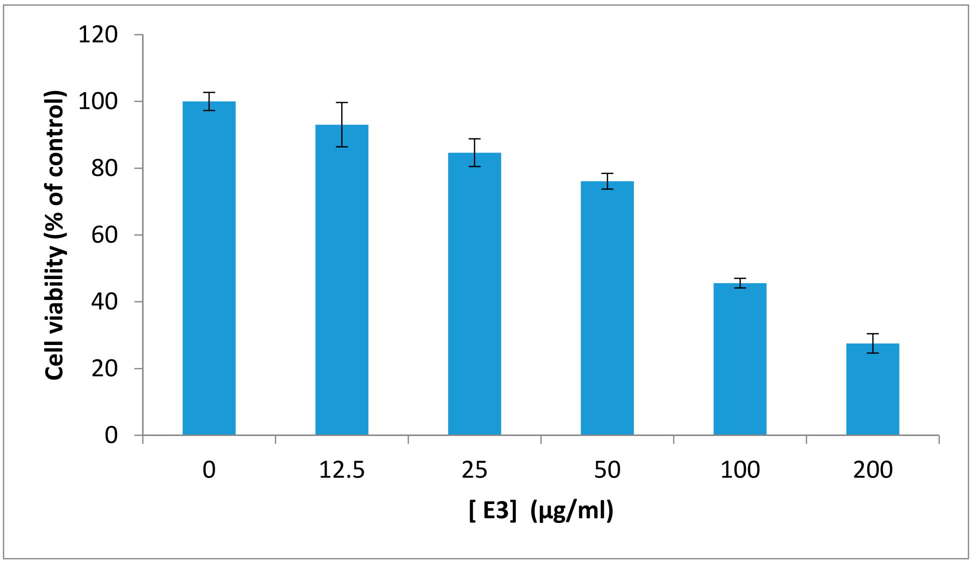


**Figure S-2.7:** Cytotoxicity of **3c** against HeLa cells

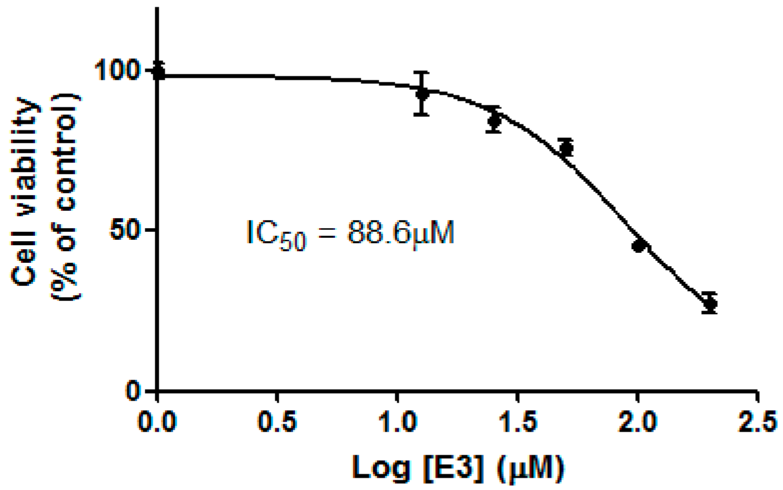


**Figure S-2.8:** Dose response curve of **3c** against HeLa cells

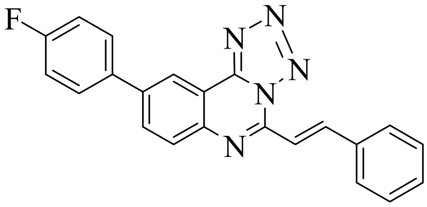

**4a**

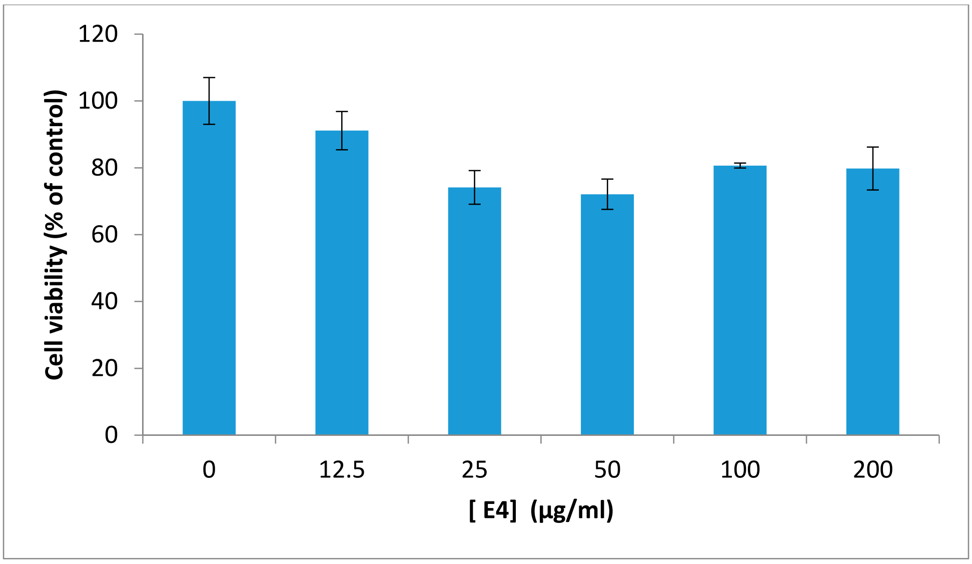


**Figure S-2.9:** Cytotoxicity of **4a** against HeLa cells

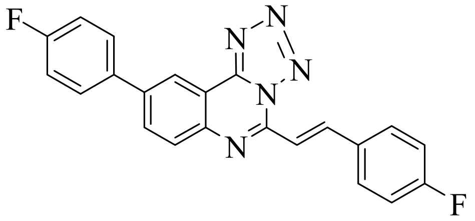


**4b**

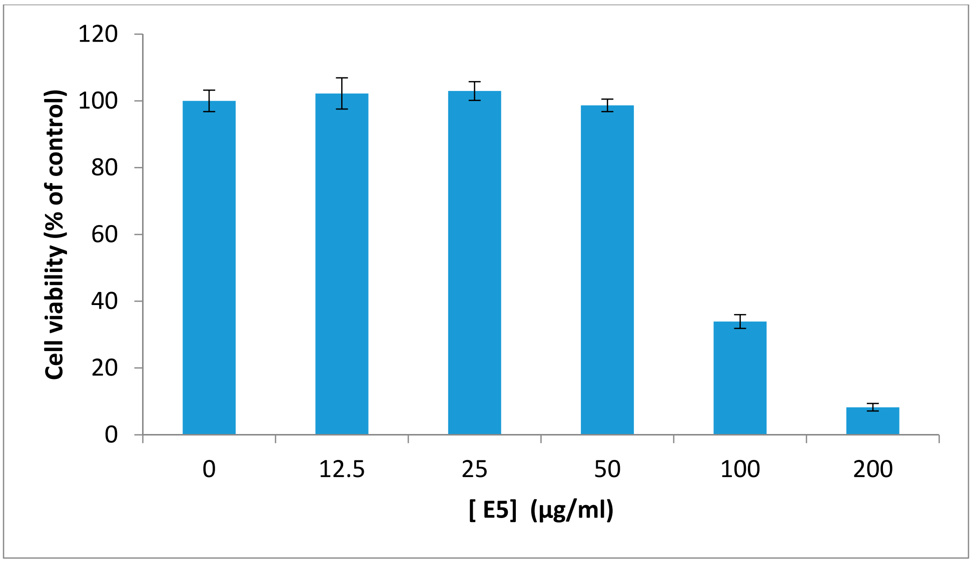


**Figure S-2.10:** Cytotoxicity of **4b** against HeLa cells

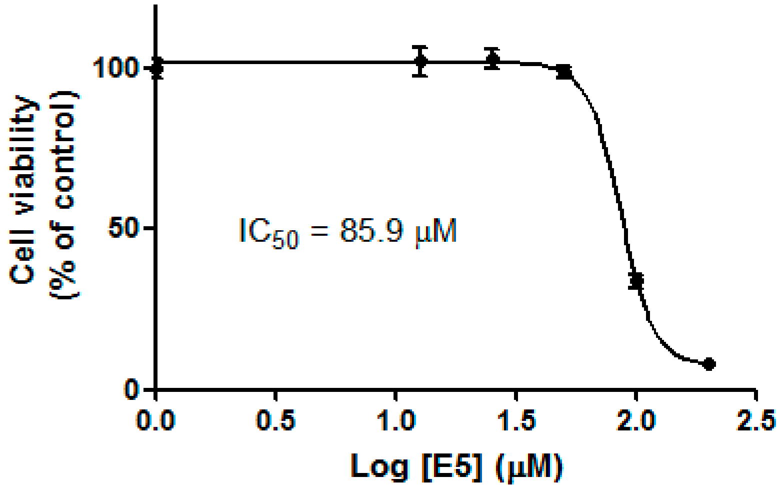


**Figure S-2.11:** Dose response curve of **4b** against HeLa cells

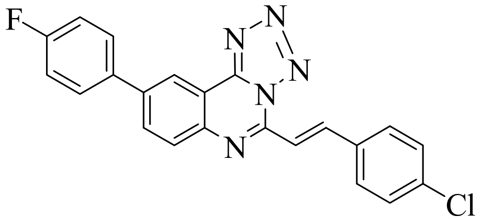

**4c**

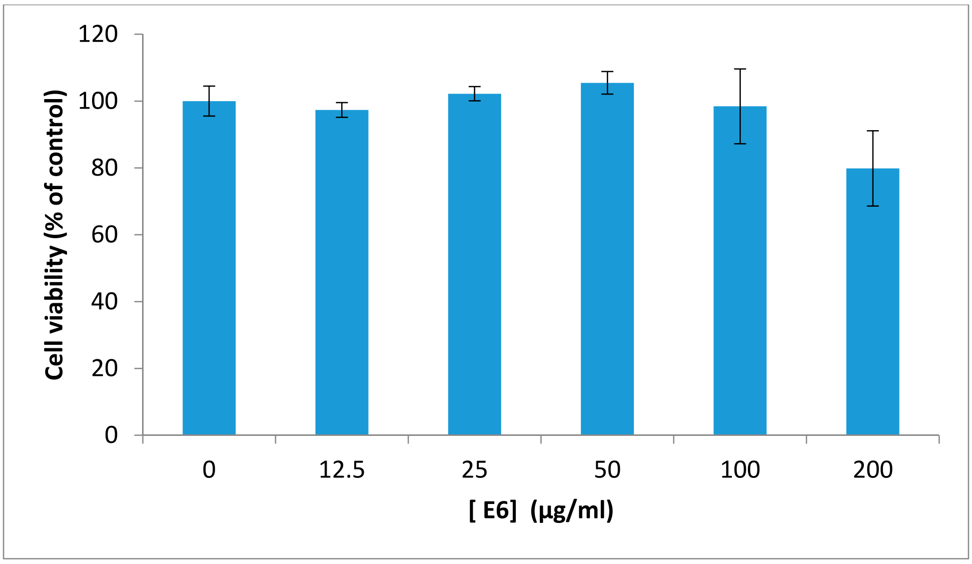


**Figure S-2.12:** Cytotoxicity of **4c** against HeLa cells

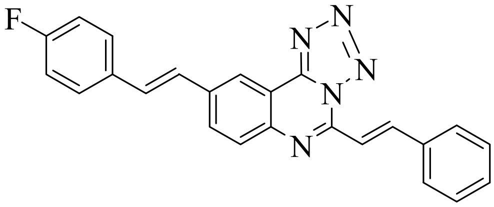


**4d**

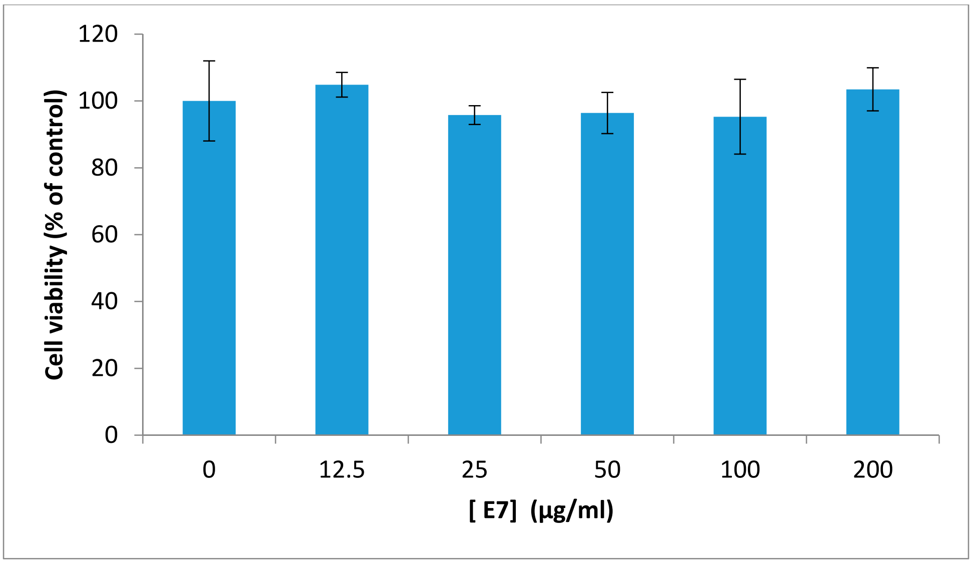


**Figure S-2.13:** Cytotoxicity of **4d** against HeLa cells

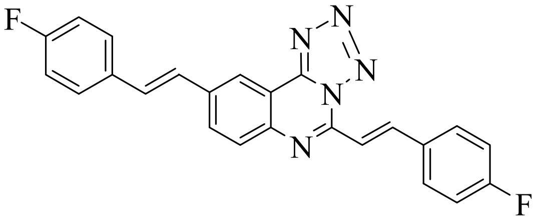


**4e**

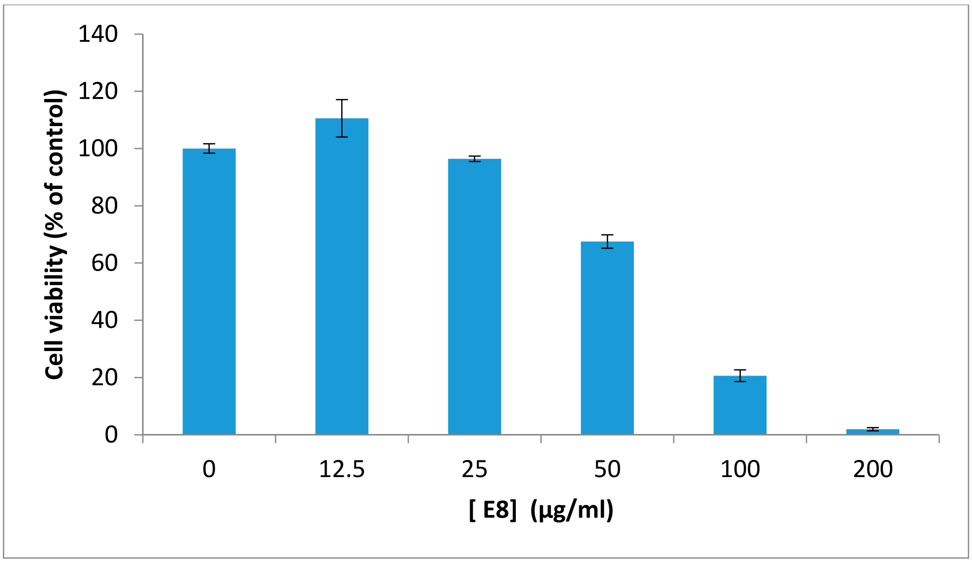


**Figure S-2.14:** Cytotoxicity of **4e** against HeLa cells

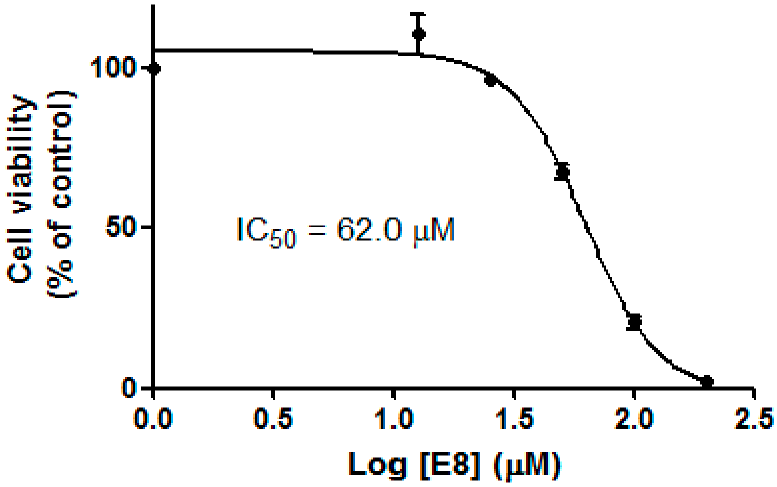


**Figure S-2.15:** Dose response curve of **4e** against HeLa cells

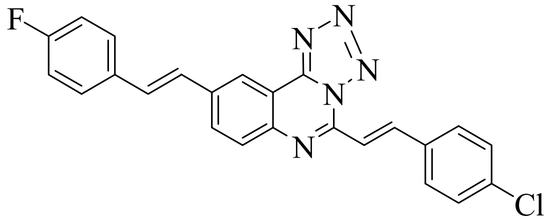

**4f**

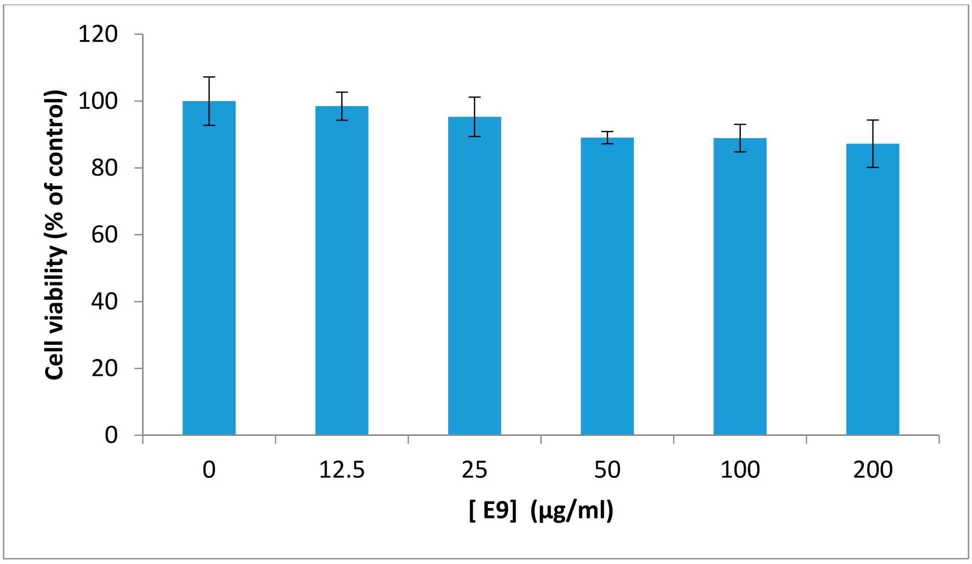


**Figure S-2.16:** Cytotoxicity of **4f** against HeLa cells

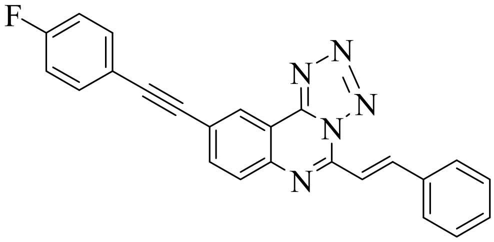


**5a**

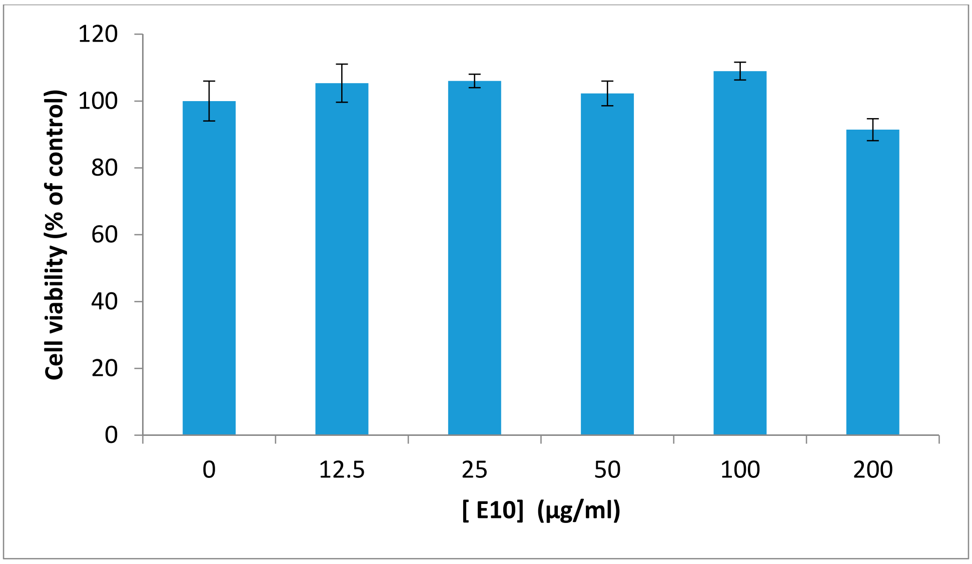


**Figure S-2.17:** Cytotoxicity of **5a** against HeLa cells

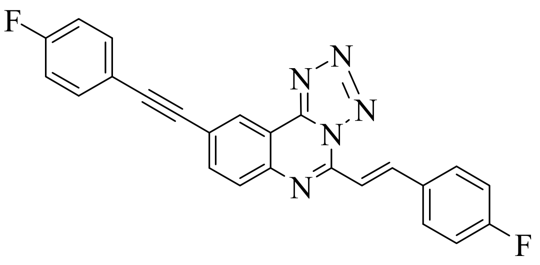


**5b**

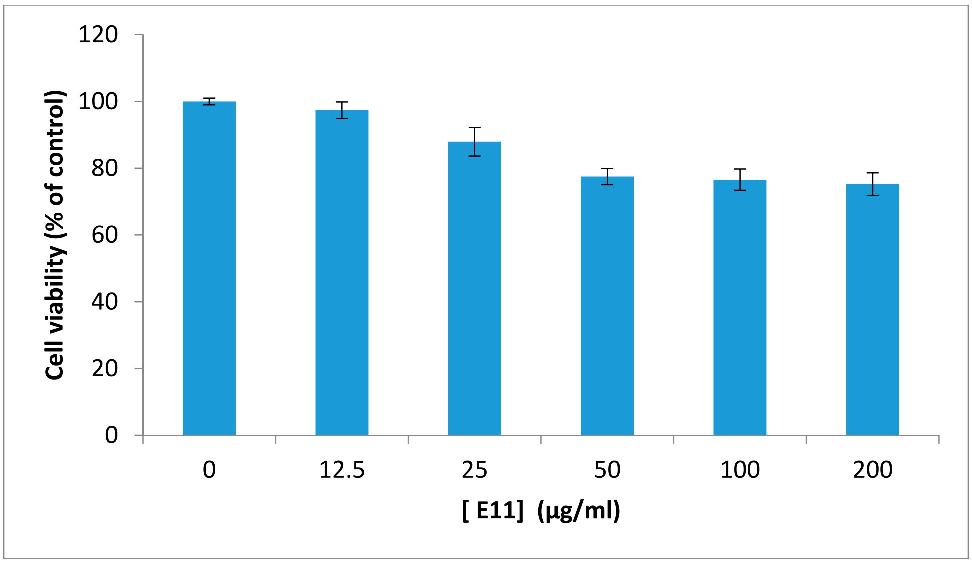


**Figure S-2.18:** Cytotoxicity of **5b** against HeLa cells

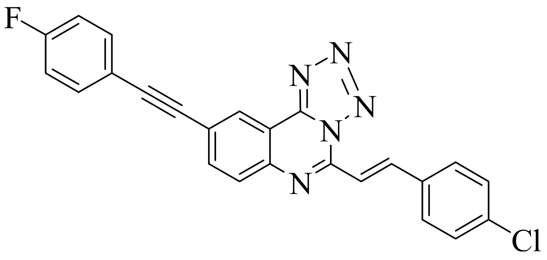


**5c**

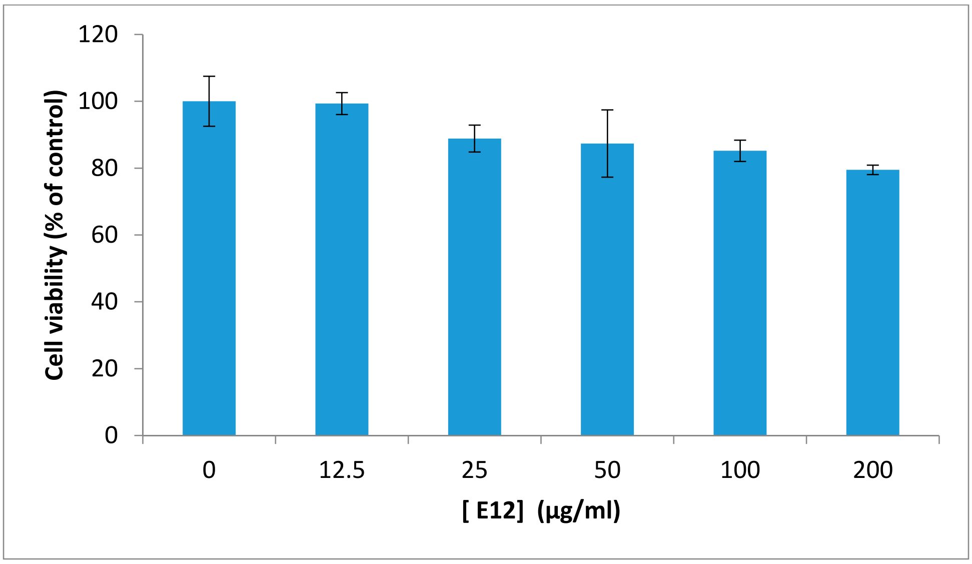


**Figure S-2.19:** Cytotoxicity of **5c** against HeLa cells

**S3: Cytotoxicity and dose response of Melphalan and compounds 3–5 curves against MCF7 cells**

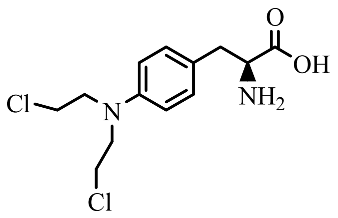


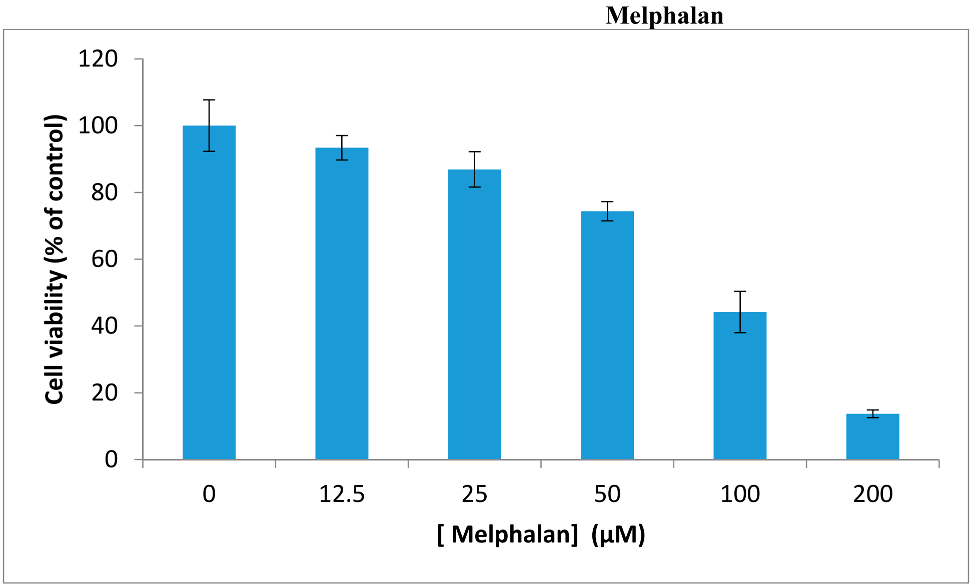


**Figure S-3.1:** Cytotoxicity of **Melphalan** against MCF-7 cells

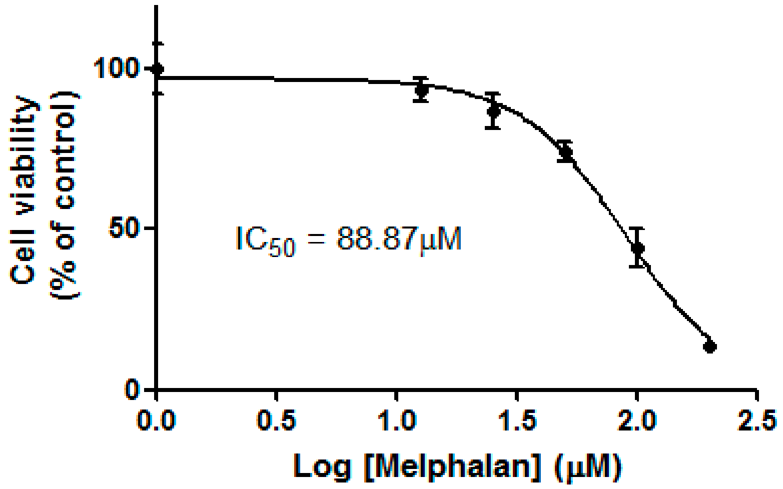


**Figure S-3.2:** Dose response curve of **Melphalan** against MCF-7 cells

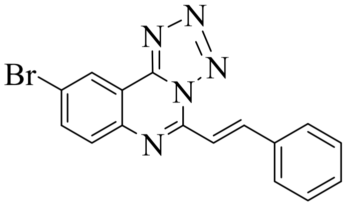

**3a**

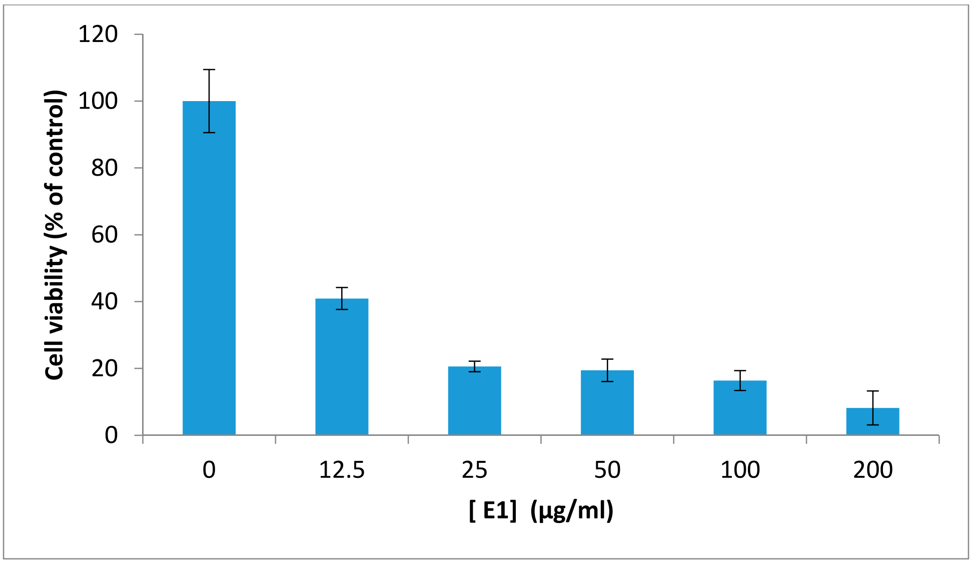


**Figure S-3.3:** Cytotoxicity of **3a** against MCF-7 cells

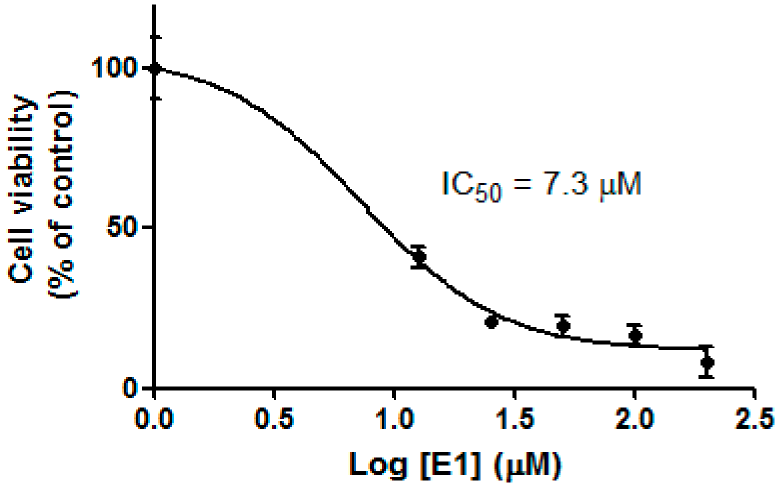


**Figure S-3.4:** Dose response curve of **3a** against MCF-7 cells

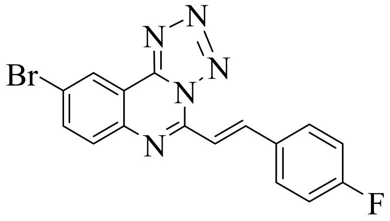


**3b**

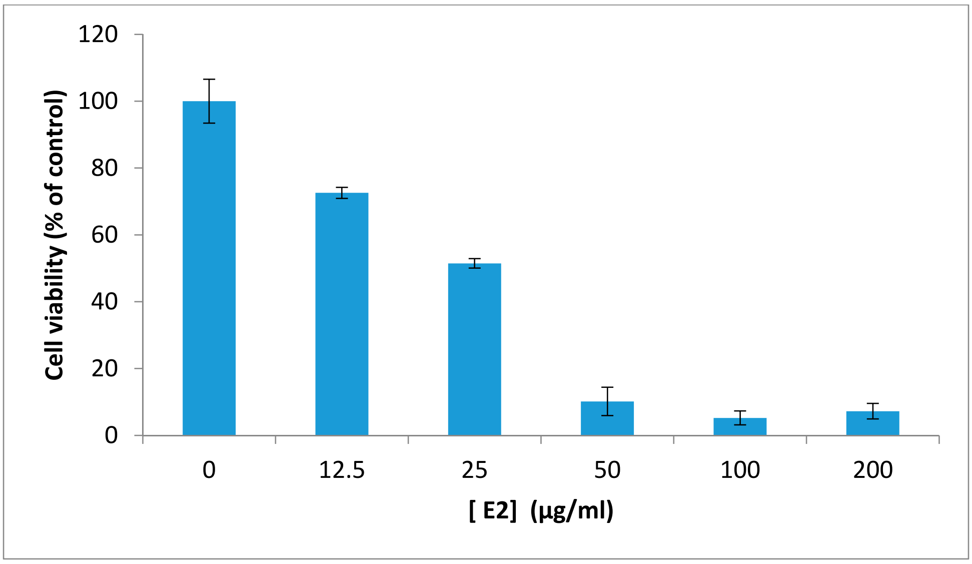


**Figure S-3.5:** Cytotoxicity of **3b** against MCF-7 cells

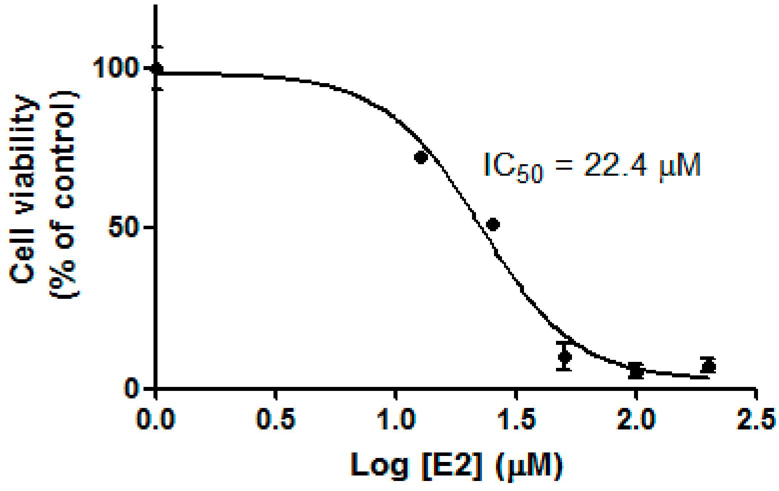


**Figure S-3.6:** Dose response curve of **3b** against MCF-7 cells

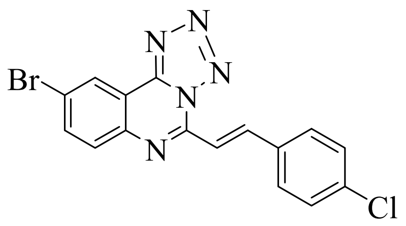


**3c**

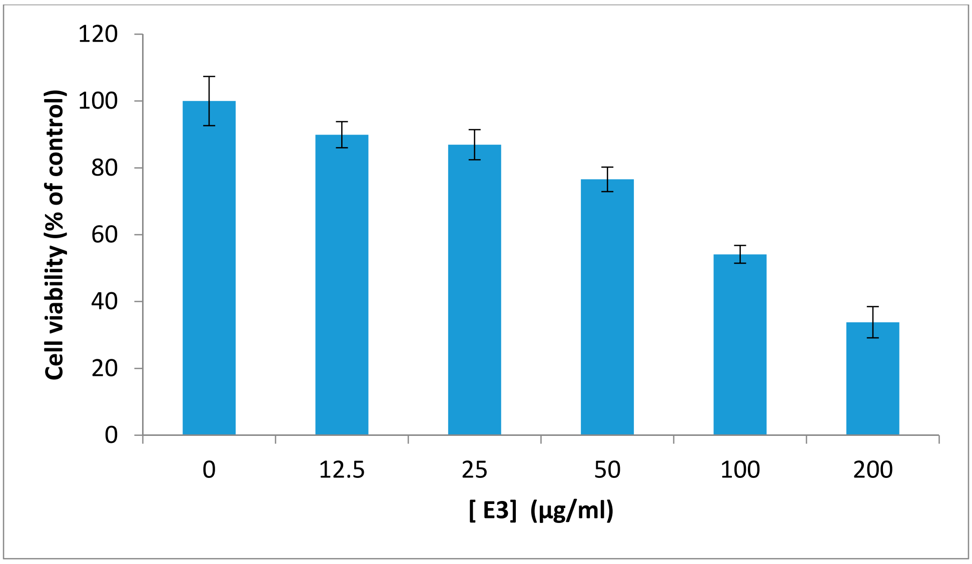


**Figure S-3.7:** Cytotoxicity of **3c** against MCF-7 cells

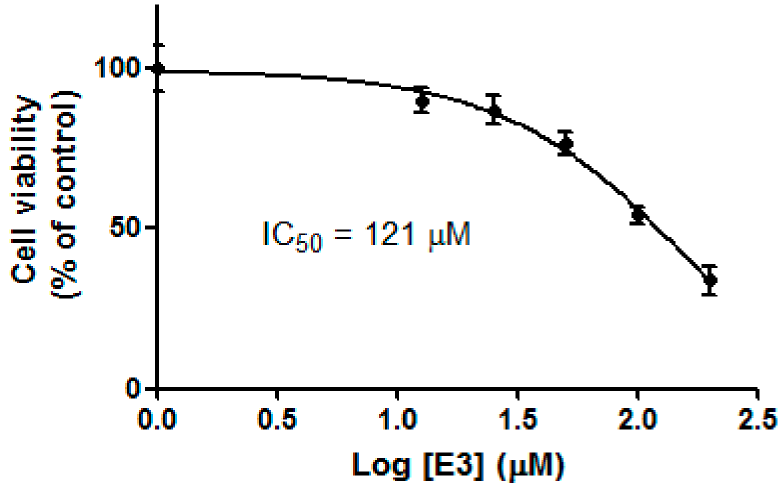


**Figure S-3.8:** Dose response curve of **3c** against MCF-7 cells

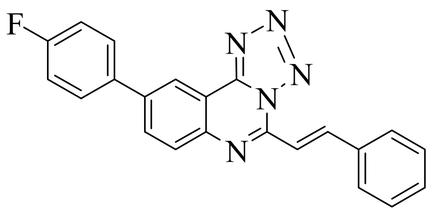


**4a**

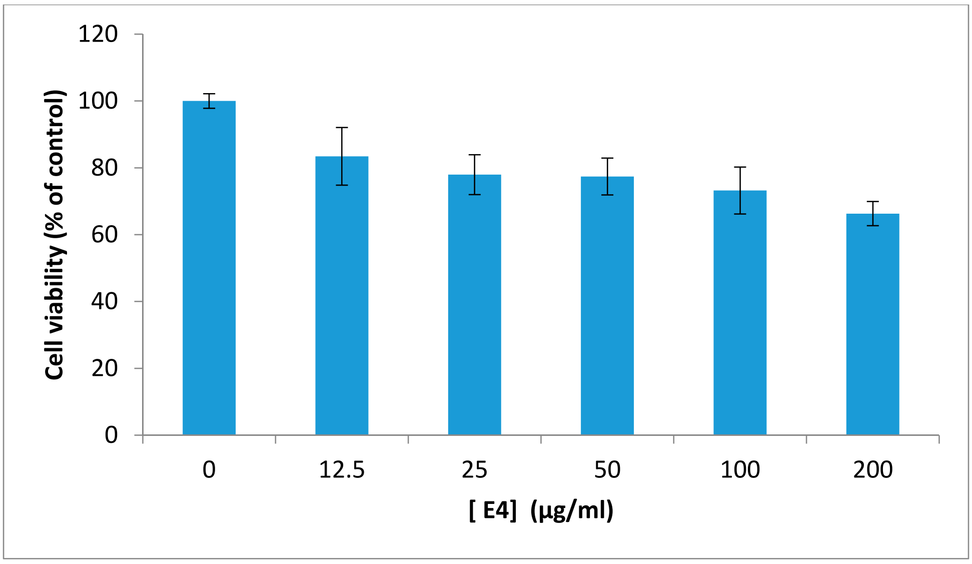


**Figure S-3.9:** Cytotoxicity of **4a** against MCF-7 cells

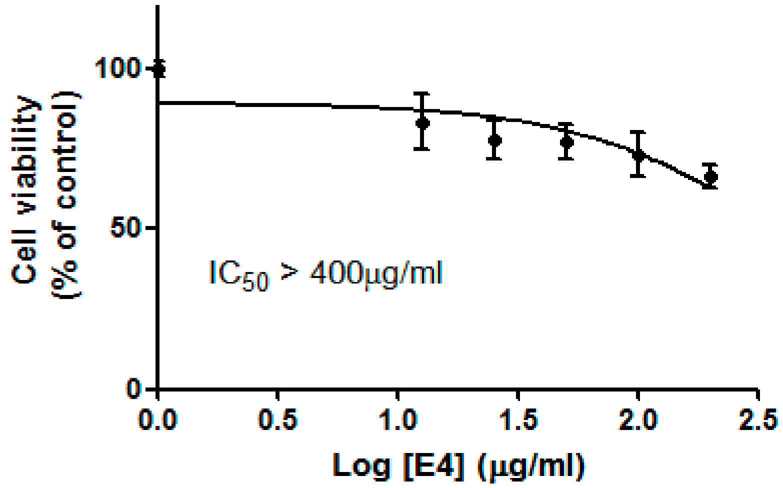


**Figure S-3.10:** Dose response curve of **4a** against MCF-7 cells

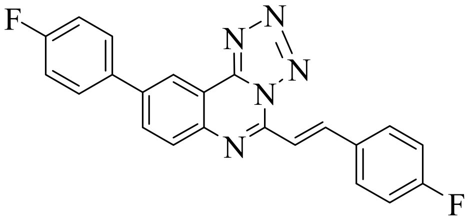

**4b**

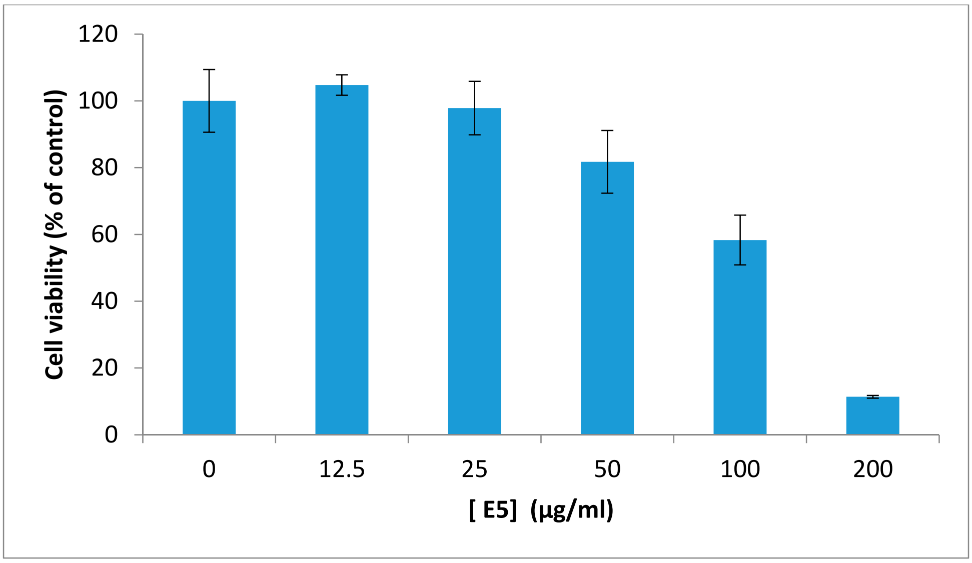


**Figure S-3.11:** Cytotoxicity of **4b** against MCF-7 cells

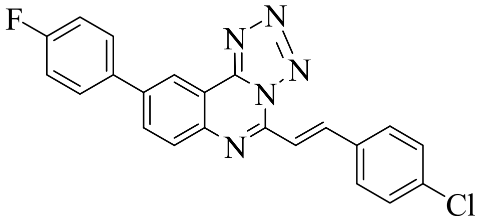


**4c**

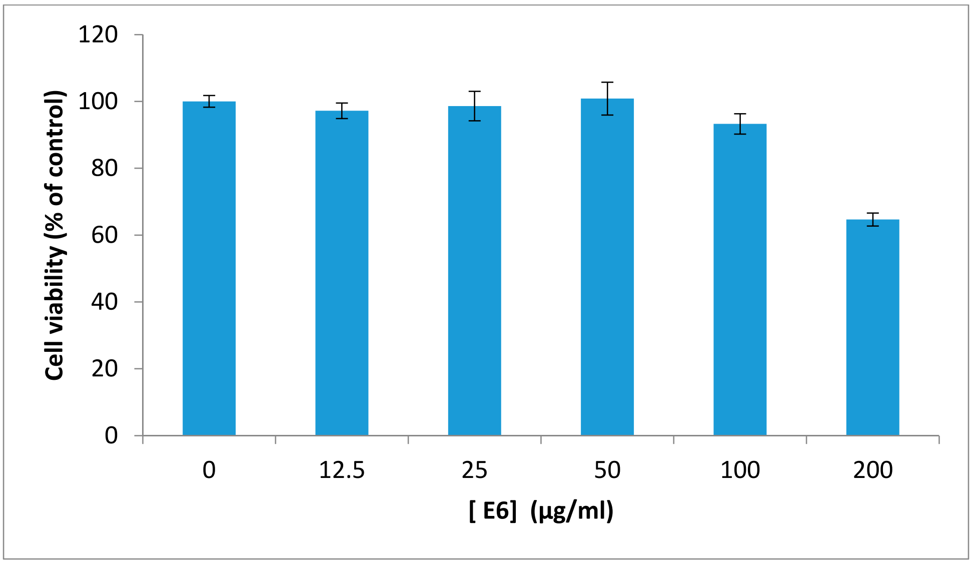


**Figure S-3.12:** Cytotoxicity of **4c** against MCF-7 cells

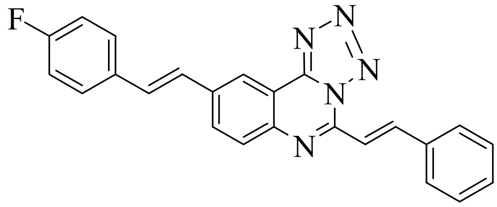

**4d**

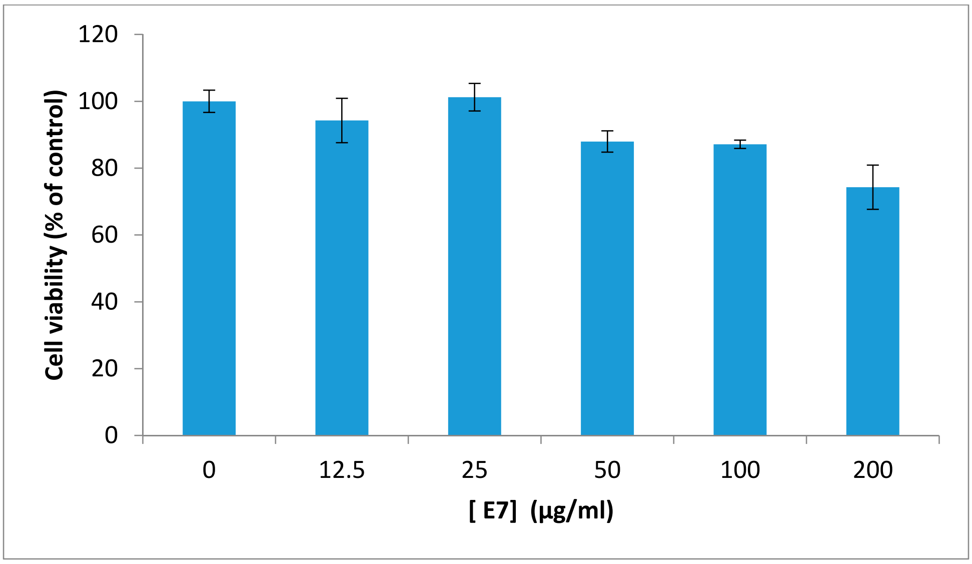


**Figure S-3.13:** Cytotoxicity of **4e** against MCF-7 cells

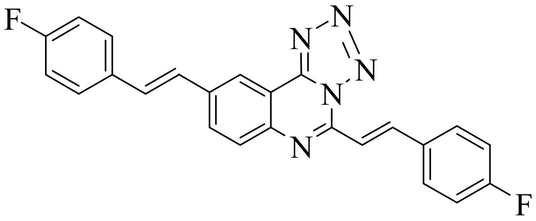


**4e**

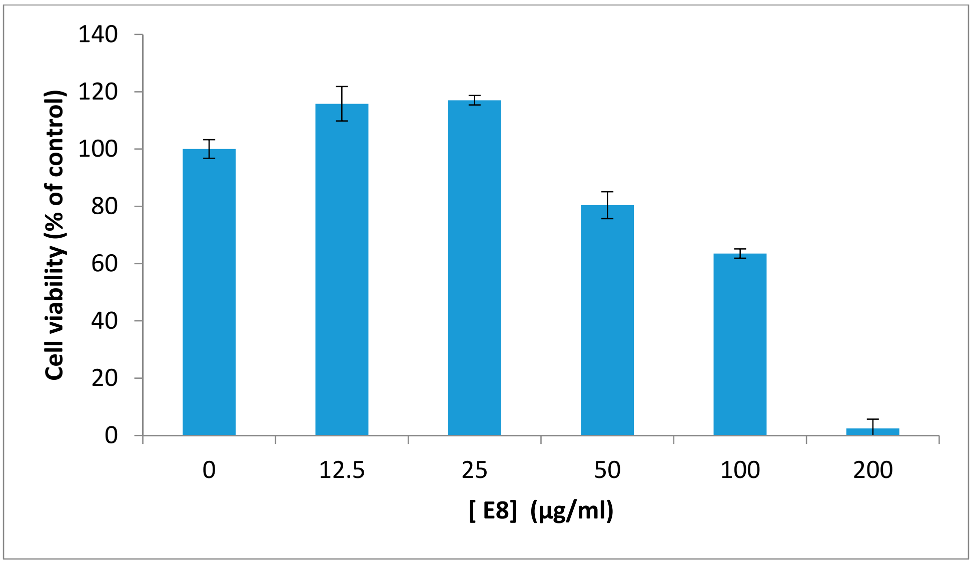


**Figure S-3.14:** Cytotoxicity of **4e** against MCF-7 cells

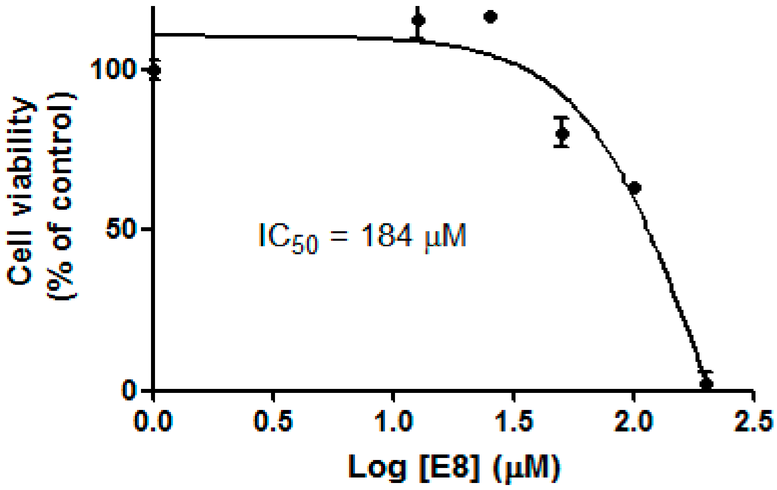


**Figure S-3.15:** Dose response curve of **4e** against MCF-7 cells

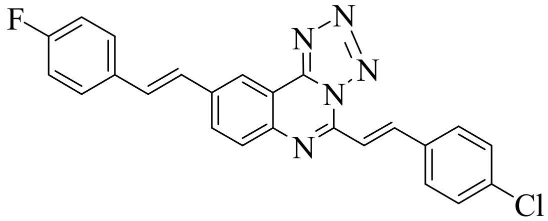


**4f**

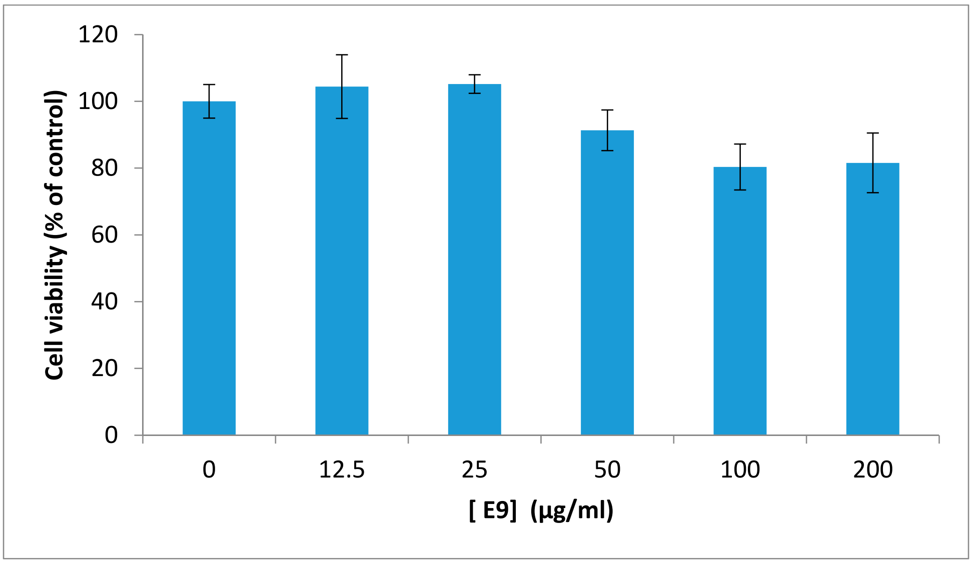


**Figure S-3.16:** Cytotoxicity of **4f** against MCF-7 cells

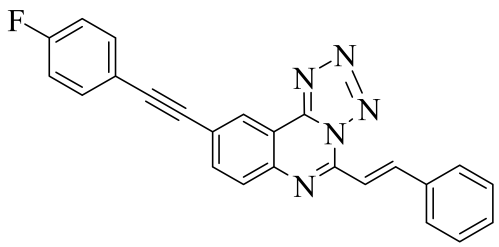

**5a**

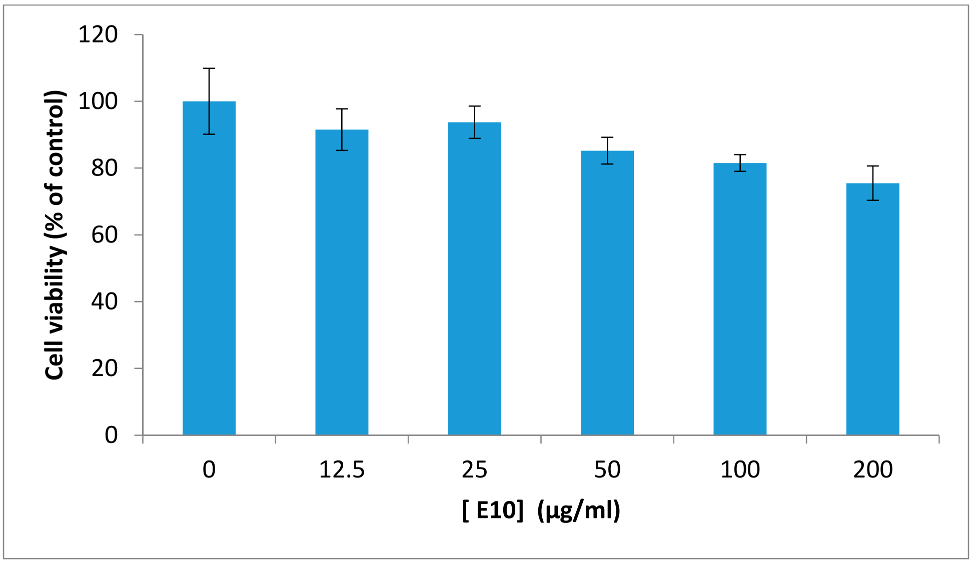


**Figure S-3.17:** Cytotoxicity of **5a** against MCF-7 cells

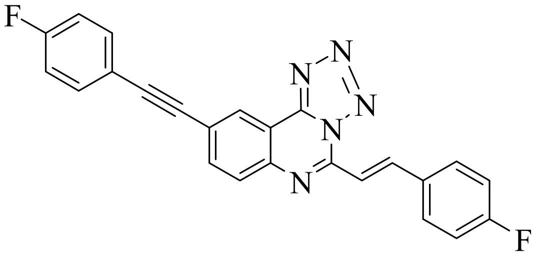

**5b**

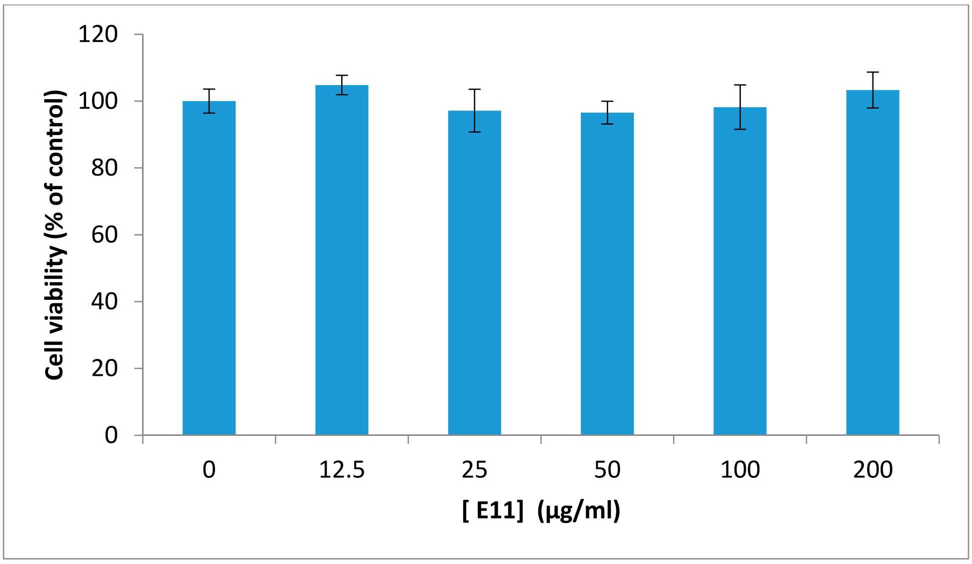


**Figure S-3.18: Cytotoxicity of 5b against HeLa cells**

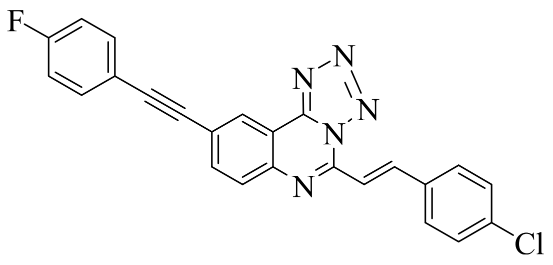


**5c**

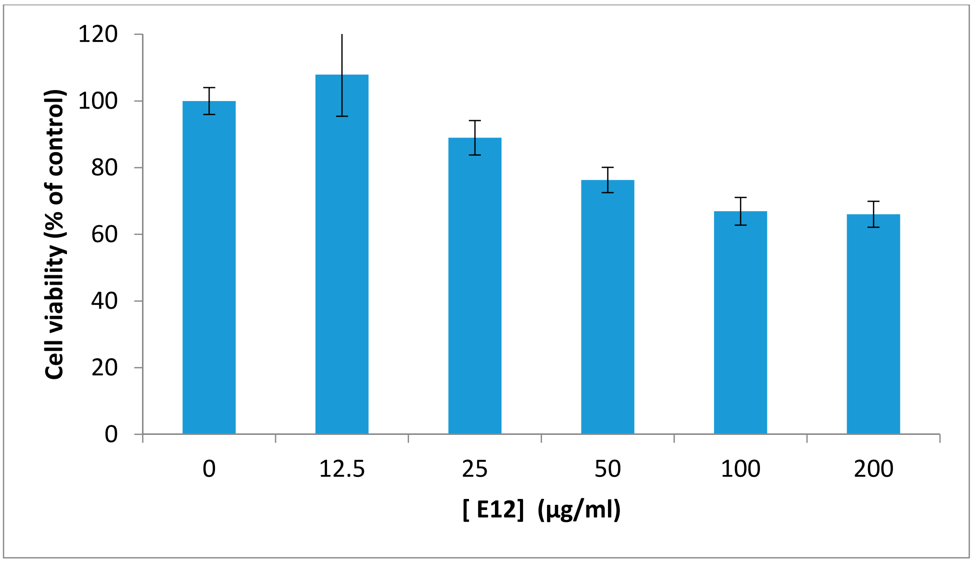


**Figure S-3.19: Cytotoxicity of 5c against HeLa cells**

**S4: Spread sheet for statistical analysis which contains p values for each test**

| Compound number | Sample Code |
|---|---|
| 3a | E1 |
| 3b | E2 |
| 3c | E3 |
| 4a | E4 |
| 4b | E5 |
| 4c | E6 |
| 4d | E7 |
| 4e | E8 |
| 4f | E9 |
| 5a | E10 |
| 5b | E11 |
| 5c | E12 |

UNISA Screening
HeLa and MCF7.xlsx
